# eRNA profiling uncovers the enhancer landscape of oesophageal adenocarcinoma and reveals new deregulated pathways

**DOI:** 10.7554/eLife.80840

**Published:** 2023-02-20

**Authors:** Ibrahim Ahmed, Shen-Hsi Yang, Samuel Ogden, Wei Zhang, Yaoyong Li, The OCCAMs consortium, Andrew D Sharrocks

**Affiliations:** 1 School of Biological Sciences, Faculty of Biology, Medicine and Health, University of Manchester Manchester United Kingdom; Dana-Farber Cancer Institute United States; Memorial Sloan Kettering Cancer Center United States

**Keywords:** oesophageal adenocarcinoma, enhancer, chromatin, eRNA, Human

## Abstract

Cancer is driven by both genetic and epigenetic changes that impact on gene expression profiles and the resulting tumourigenic phenotype. Enhancers are transcriptional regulatory elements that are key to our understanding of how this rewiring of gene expression is achieved in cancer cells. Here, we have harnessed the power of RNA-seq data from hundreds of patients with oesophageal adenocarcinoma (OAC) or its precursor state Barrett’s oesophagus coupled with open chromatin maps to identify potential enhancer RNAs and their associated enhancer regions in this cancer. We identify ~1000 OAC-specific enhancers and use these data to uncover new cellular pathways that are operational in OAC. Among these are enhancers for *JUP*, *MYBL2*, and *CCNE1*, and we show that their activity is required for cancer cell viability. We also demonstrate the clinical utility of our dataset for identifying disease stage and patient prognosis. Our data therefore identify an important set of regulatory elements that enhance our molecular understanding of OAC and point to potential new therapeutic directions.

## Introduction

Enhancers are distal regulatory elements that generally promote gene expression by engaging with the promoters of their target genes in cis (Andersson and Sandelin, 2020), although they have also been observed acting in trans (Hu et al., 2008; Spilianakis et al., 2005) and more recently, through hubs of activity on extra-chromosomal DNA species (Hung et al., 2021). Active enhancers are characterised by the presence of histone marks such as H3K27ac and H3K4me1 (Creyghton et al., 2010; Heintzman et al., 2007). However, it has been shown that enhancers can be the site of production for small transcripts termed enhancer RNAs (eRNAs) (De Santa et al., 2010; Kim et al., 2010). Whilst the functionality of eRNAs is still under debate, there is a large body of evidence associating the production of eRNAs with enhancer activity, and subsequent target gene activation (Tyssowski et al., 2018; Andersson et al., 2014; Chen et al., 2018). This association with gene expression has allowed eRNA-defined enhancer activity to serve as a specific marker for developmental stage and tissue type (Yan et al., 2019; Huang et al., 2016). Furthermore, eRNAs provide molecular markers for disease, often with more sensitivity than the gene expression pattern itself (Zhang et al., 2019; Chen et al., 2018).

During tumourigenesis, there are widespread changes to gene expression patterns that are associated with rewiring of the regulatory landscape in an enhancer-driven manner (Li et al., 2015; Hsieh et al., 2014). This can be accompanied by eRNA production. For example, production of an eRNA from the *PSA* gene enhancer is associated with increased *PSA* expression in castration-resistant prostate cancer (Zhao et al., 2016). This potentially makes the production of eRNAs a biomarker for cancer, but this is widely underappreciated. Indeed, a recent pan cancer study demonstrated that eRNAs can serve as prognostic markers across various cancer types and provide novel insights into cancer biology (Chen et al., 2018), leading to the identification of therapeutic opportunities.

Oesophageal adenocarcinoma (OAC) has an overall 5-year survival rate of approximately 15%, making it a leading global cause of cancer-associated deaths (Coleman et al., 2018). OAC is believed to arise in a stepwise fashion from the pre-cancerous lesion Barrett’s oesophagus (BO) (Peters et al., 2019). A number of large-scale DNA sequencing studies have been performed into the pathogenesis of OAC from Barrett’s (Frankell et al., 2019; Ross-Innes et al., 2015; Stachler et al., 2015), however there is still uncertainty concerning the precise molecular mechanisms. In an effort to understand potential epigenetic contributors to OAC, we have previously demonstrated that changes in chromatin accessibility play a role in the transition to OAC (Britton et al., 2017; Rogerson et al., 2019; Rogerson et al., 2020). These chromatin changes are often associated with non-coding regions of the genome that may represent regulatory elements such as enhancers.

Here, we build on our previous work identifying chromatin changes during the BO to OAC transition (Britton et al., 2017; Rogerson et al., 2019; Rogerson et al., 2020). By integrating total RNA-seq data from BO and OAC patients generated by the Oesophageal Cancer Clinical and Molecular Stratification (OCCAMS) consortium dataset, with previously generated chromatin accessibility data on these tissues (Britton et al., 2017; Rogerson et al., 2019; Rogerson et al., 2020; Corces et al., 2018), we identify, and validate, pervasive eRNA production at regions of accessible chromatin, indicative of enhancer activity. Subsequent interrogation of genes associated with these enhancers identified deregulated pathways of importance and demonstrated the potential clinical utility of eRNA profiling in OAC.

## Results

### Identification of potential intergenic eRNA transcripts in OAC patients

**Figure 1.**
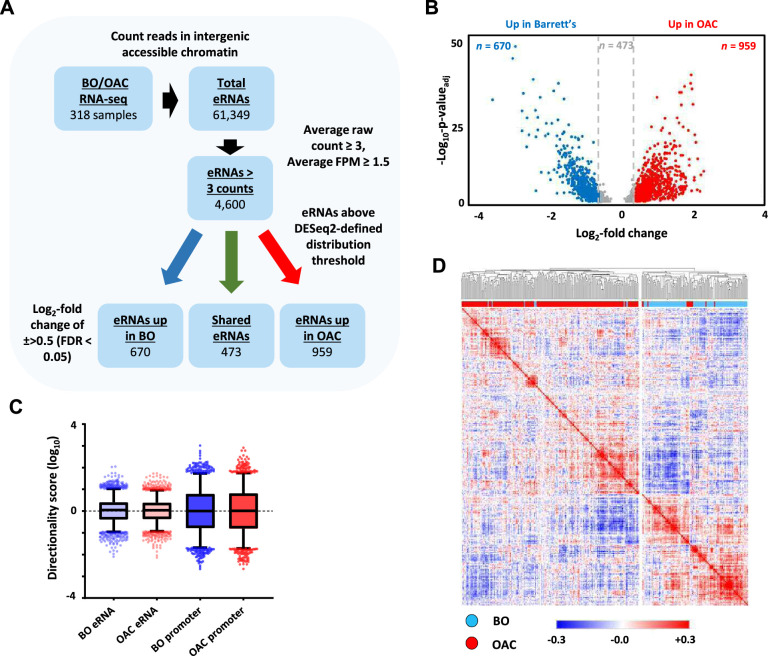
Identification of enhancer transcription in oesophageal adenocarcinoma (OAC) and Barrett’s patients. (**A**) Enhancer RNA (eRNA) identification strategy. The numbers of putative eRNAs identified at each stage are indicated. (**B**) Volcano plot displaying the differentially expressed (±Log_2_FC 0.5,<p_adj_ = 0.05) eRNAs (*n* = 2102). (**C**) Directionality scores for Barrett’s oesophagus (BO)- or OAC-specific eRNAs compared to promoters. (**D**) Pearson’s correlation and hierarchical clustering of BO (*n* = 108) and OAC (*n* = 210) patient tissue total RNA-seq samples according to row *z*-score normalised expression levels in the 4600 eRNA regions. See also Figure 1—figure supplement 1.

eRNAs are generally unstable and lowly abundant, making them hard to detect in RNA-seq datasets. We therefore harnessed the sequencing power derived from combining hundreds of patient samples to discover potential eRNAs (Figure 1A). To identify eRNAs that are relevant to OAC we interrogated RNA-seq data generated from 210 OAC patients and also from 108 Barrett’s patients to identify differentially upregulated eRNAs in OAC (Jammula et al., 2020). After mapping sequencing reads to the genome, we excluded all regions corresponding to gene bodies as well as sequences 2 kb upstream from the transcriptional start site (TSS) and 500 bp downstream from the annotated transcriptional termination site (TTS) (Figure 1—figure supplement 1A) to avoid interference from promoter sequences and read through transcription, respectively. Next, we identified all of the accessible regions of chromatin in OAC and Barrett’s samples within this truncated genome by creating a union peak set from ATAC-seq performed on 14 OAC and 4 Barrett’s samples (Britton et al., 2017; Rogerson et al., 2019; Rogerson et al., 2020; Corces et al., 2018) (resulting in 150,265 peaks). To focus on potential enhancer regions, we then took the RNA-seq data from both classes of patients and assessed raw reads within these accessible chromatin regions. This identified 61,349 intergenic regions that contain RNA transcripts and represent potential eRNA regions. We then filtered these based on a read count ≥3 and fragments per million (FPM) value ≥1.5, which resulted in a final high-confidence set of 4600 potential eRNA containing enhancer regions in OAC and Barrett’s patients (Supplementary file 1).

**Figure 1—figure supplement 1.**
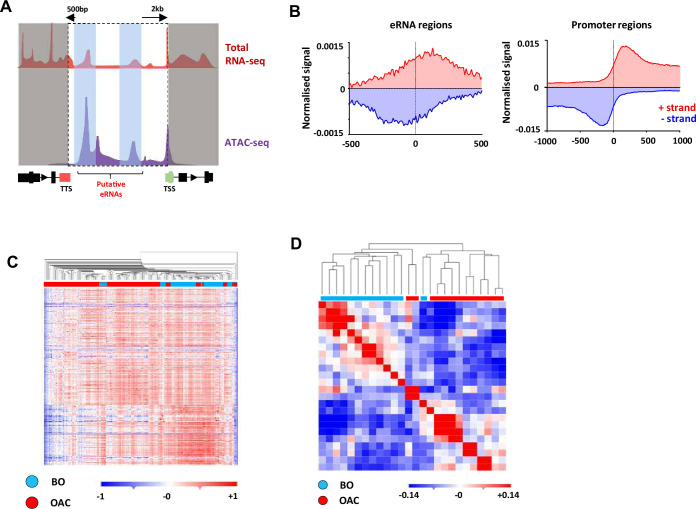
Identification of enhancer transcription in Barrett’s oesophagus (BO) and oesophageal adenocarcinoma (OAC) patients. (**A**) Diagram of the sites of transcription considered for enhancer RNA (eRNA) analysis (bracketed). Total RNA-seq reads were integrated with intergenic regions of accessible chromatin (highlighted) at least 500 bp downstream/2 kb upstream of genes. (**B**) RNA-seq signal in BO (*n* = 108) and OAC (*n* = 210) patient tissue total RNA-seq samples plotted in a strand-specific manner across a 1 kb region centred on the 2102 eRNA containing regions (left) or a 2 kb region centred on 3030 promoter regions (right). Data are normalised for total number of reads. (**C**) Pearson’s correlation and hierarchical clustering of BO (*n* = 108) and OAC (*n* = 210) patient tissue total RNA-seq samples (OCCAMs) according to row *z*-score normalised gene expression levels. (**D**) Pearson’s correlation and hierarchical clustering of BO (*n* = 13) and OAC (*n* = 13) patient tissue total RNA-seq samples (Maag et al., 2017) according to row *z*-score normalised expression levels in the 4600 eRNA regions.

Next, we identified differentially transcribed eRNAs in each disease state (±>0.5 log_2_ fold; p-value_adj_ <0.05; Figure 1B) and found 959 to be significantly upregulated in OAC (Supplementary file 2) and 670 to be more active in Barrett’s patients (Supplementary file 3). 2498 eRNAs did not meet the stringent DESeq2 distribution threshold and were discarded. The remaining putative eRNAs exhibited low directionality score distributions consistent with the bidirectional transcription associated with eRNA production (Figure 1C; Figure 1—figure supplement 1B).

To probe the clinical utility of eRNA region profiling, we analysed the expression levels found in the 4600 eRNA regions across all of the OAC and Barrett’s samples. Hierarchical clustering showed a clear separation of Barrett’s and OAC patients (Figure 1D). While clustering based on whole RNA-seq data gave broadly similar separation of OAC and Barrett’s samples, several more were misclassified compared to eRNA-based profiling (Figure 1—figure supplement 1C; 37 vs. 8 samples). Clustering of the expression of the same eRNA regions in a different RNA-seq dataset (Maag et al., 2017) also provided a good separation of the OAC and Barrett’s samples (Figure 1—figure supplement 1D), further demonstrating the relevance of this dataset. In summary therefore, we have identified a panel of potential eRNA generating regions that can be used for discriminating OAC samples from the pre-cancerous Barrett’s state.

### eRNA-associated regions show enhancer-like characteristics

**Figure 2.**
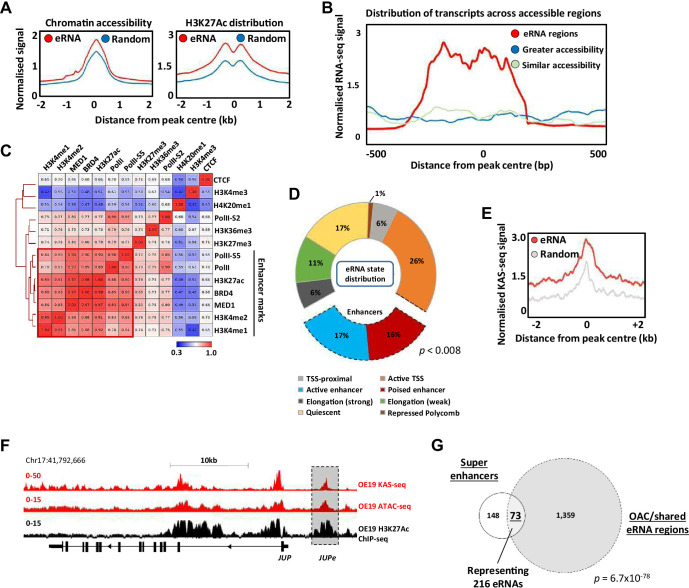
Putative enhancer RNAs (eRNAs) are associated with enhancer-like genomic regions. (**A**) Metaplots of patient tissue chromatin accessibility (left) and OE19 cell H3K27ac ChIP-seq signal (right) at all 4600 eRNA regions compared to 4600 random regions of accessible chromatin. (**B**) Distribution of transcription at 4600 eRNA regions compared to 4600 randomly selected regions of similar or greater chromatin accessibility (regions shown as peak centre ±0.5 kb). (**C**) Pearson’s correlation and hierarchical clustering of CUT&Tag signal at 4600 eRNA regions for various chromatin-associated factors. (**D**) Distribution of ChromHMM emission states for 4600 eRNA regions. (**E**) Metaplots of KAS-seq signal in OE19 cells at 4600 eRNA regions compared to 4600 random regions of accessible chromatin. (**F**) Genome browser view of OE19 KAS-seq, OE19 ATAC-seq data, and OE19 H3K27ac ChIP-seq at the *JUP* locus with the *JUPe* eRNA highlighted. (**G**) Venn diagram of overlap between 221 high-confidence intergenic super enhancers and 1432 eRNAs (specific to oesophageal adenocarcinoma [OAC] or shared with Barrett’s oesophagus (BO) eRNA; p-value is shown; hypergeometric test). See also Figure 2—figure supplements 1–3.

Having identified a group of accessible chromatin regions expressing potential eRNAs, we next sought further evidence to associate these with enhancer-like activity. First, we examined chromatin accessibility in OAC patients and found higher levels at eRNA expressing loci compared to a random set of open chromatin regions (Figure 2A; left). Furthermore, these regions also exhibited higher levels of the H3K27ac chromatin mark in OE19 OAC-derived cells that is usually associated with active enhancers (Figure 2A; right). This was not just a function of increased accessibility as a control group of more highly accessible regions did not exhibit increased levels of H3K27ac (Figure 2—figure supplement 1A). Indeed, chromatin accessibility levels are a weak indicator of eRNA transcription levels across patient samples, as even regions of greater accessibility contained much lower transcript levels (Figure 2B; Figure 2—figure supplement 1B). Thus, eRNA regions are more accessible but the reciprocal is not true, as more accessible regions do not necessarily show higher levels of eRNA transcription.

**Figure 2—figure supplement 1.**
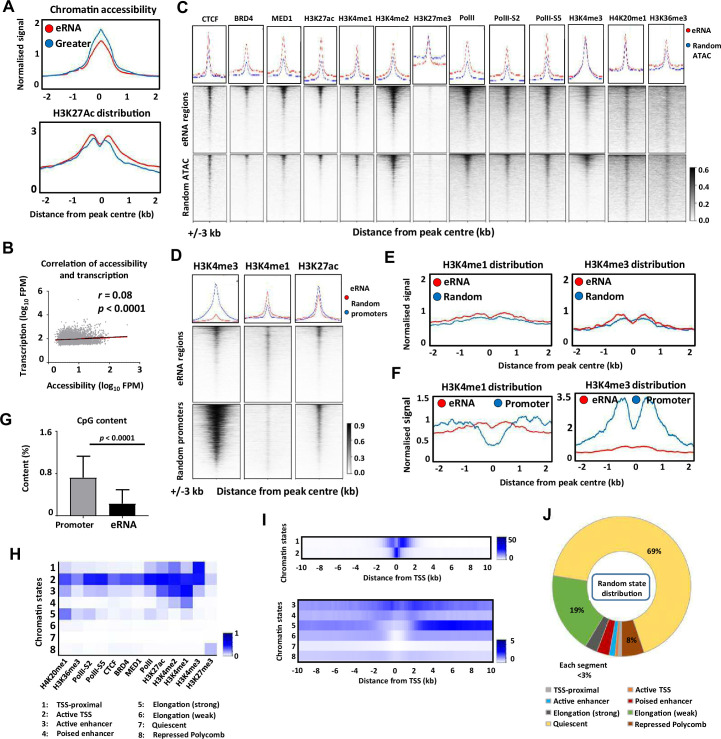
Putative enhancer RNAs (eRNAs) are associated with enhancer-like genomic regions. (**A**) Metaplots of patient tissue chromatin accessibility and OE19 cell H3K27ac ChIP-seq signal at 4600 eRNA regions, compared to 4600 random regions of accessible chromatin of greater accessibility level. (**B**) Correlation of accessibility and transcription at 4600 eRNA regions in Barrett’s oesophagus (BO, *n* = 4) and oesophageal adenocarcinoma (OAC, *n* = 14) patient tissue ATAC-seq samples and BO (*n* = 108) and OAC (*n* = 210) patient tissue total RNA-seq samples (Spearman’s *r* and p-value are shown; Spearman’s rank correlation test). (**C**) Metaplots (top panels) and heatmaps showing CUT&TAG signal for the indicated marks in OE19 cells at 4600 eRNA regions (middle panels) compared against 4600 randomly selected regions of accessible chromatin (bottom panels). (**D**) Metaplots (top panels) and heatmaps showing CUT&TAG signal in OE19 cells at 4600 eRNA regions (middle panels) compared against 4600 randomly selected accessible promoters (bottom panels). Heatmaps for H3K4me3, H3K4me1, and H3K27ac are shown. (All regions are shown as peak centre ±3 kb.) (**E**) Metaplots of H3K4me1 (left) and H3K4me3 (right) gastric adenocarcinoma (GAC) patient tissue ChIP-seq signal at 4600 eRNA regions compared to 4600 randomly selected region of accessible chromatin (regions shown as peak centre ±2 kb). (**F**) Metaplots of H3K4me1 (left) and H3K4me3 (right) GAC patient tissue ChIP-seq signal at all 4600 eRNA regions compared to 4600 randomly selected accessible promoters (all regions shown as peak centre ±2 kb). (**G**) Bar graph displaying CpG content at eRNAs versus randomly selected accessible promoters (p-value is shown; Kolmogorov–Smirnov test). (**H**) Heatmap of chromatin states generated from CUT&TAG data using ChromHMM. (**I**) Heatmaps displaying distance from transcriptional start site (TSS) for chromatin states 1 and 2 (top), and 3–8 (bottom). (**J**) Distribution of ChromHMM chromatin states for 4600 random regions of the genome.

**Figure 2—figure supplement 2.**
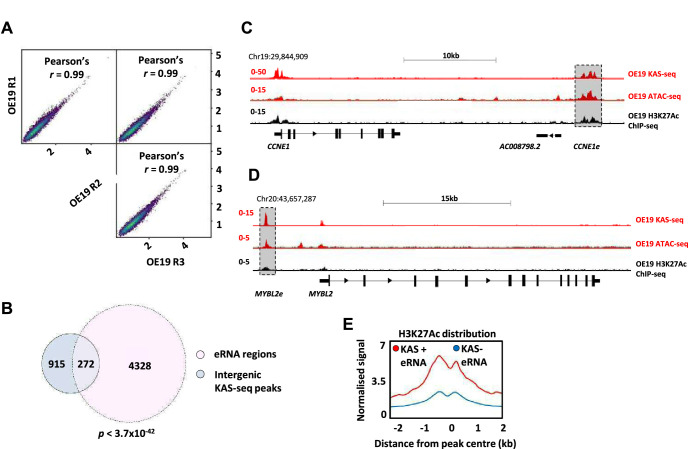
KAS-seq reveals active transcription at enhancer RNA (eRNA) regions. (**A**) Scatter plots displaying correlation of OE19 KAS-seq biological replicates (Pearson’s *r* is shown). (**B**) Venn diagram of overlap between 4600 eRNA regions and intergenic OE19 KAS-seq peaks (p-value is shown; hypergeometric test). Genome browser views of OE19 KAS- and ATAC-seq data, and OE19 H3K27ac ChIP-seq at the *CCNE1* (**C**) and *MYBL2* (**D**) loci with the *CCNE1e* and *MYBL2* eRNAs highlighted. (**E**) Metaplots of H3K27ac ChIP-seq signal in OE19 cells at 272 KAS + eRNA regions compared to 4328 KAS − eRNA regions.

**Figure 2—figure supplement 3.**
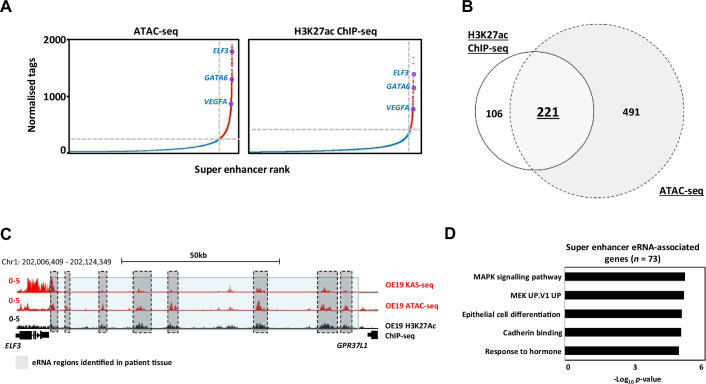
Enhancer RNAs (eRNAs) are associated with super enhancers. (**A**) Scatter plots displaying super enhancer stitching on OE19 H3K27Ac ChIP-seq (left) and OE19 ATAC-seq (right) using HOMER. (**B**) Venn diagram of overlap between super enhancers identified in OE19 ATAC-seq and OE19 H3K27Ac ChIP-seq. (**C**) Genome browser view of KAS-seq, ATAC-seq data, and H3K27ac ChIP-seq in OE19 cells and eRNAs detected in oesophageal adenocarcinoma (OAC) patient samples at the *ELF3* locus, with the *ELF3* super enhancer highlighted. Putative enhancer regions defined by eRNA expression in OAC patient tissue are shaded. (**D**) Gene ontology (GO)-term analysis of genes annotated to high-confidence super enhancers associated with eRNAs.

To provide further evidence for association with active enhancers in OAC cells, we used CUT&Tag (Kaya-Okur et al., 2020) to profile a range of histone marks and chromatin-associated proteins in OE19 cells and correlated the levels of these across the eRNA expressing regions (Figure 2C). There is clear co-association with a range of enhancer-associated marks and proteins, including RNA polymerase II, BRD4, and the MED1 subunit of mediator. This is also evident when visualising the data as heatmaps compared to random regions of accessible chromatin, where there are higher levels of the chromatin-associated marks/proteins that categorise enhancers in the eRNA regions (Figure 2—figure supplement 1C). Conversely, there is a clear depletion of the promoter-associated mark H3K4me3 (Figure 2—figure supplement 1D). We also examined the distribution of the chromatin marks H3K4me1 (associated with active and poised enhancers) and H3K4me3 (associated with active promoters) found in gastric adenocarcinoma (GAC) patients which are molecularly similar to OAC patients (Cancer Genome Atlas Research Network et al., 2017). Neither mark is enriched in the eRNA containing regions compared to random accessible regions (Figure 2—figure supplement 1E). However, there is a clear enrichment of H3K4me1 and depletion of H3K4me3 in eRNA containing regions compared to promoters, consistent with their designation as potential enhancers (Figure 2—figure supplement 1F). Conversely, the CpG content of the eRNA regions was substantially lower than at promoter regions (Figure 2—figure supplement 1G).

To further probe links between eRNA containing regions and enhancer activity, we partitioned the genome into a series of states via a Hidden Markov Model (HMM states) (Figure 2—figure supplement 1H). Positional information was used to mark these HMM states as promoter proximal or distal (Figure 2—figure supplement 1I). Re-evaluation of the eRNA expressing regions showed that 33% were associated with regions designated as enhancers (Figure 2D; compared to 4% genome-wide), with very few regions designated as quiescent or repressed (17% compared to 69% in randomly selected chromatin regions; Figure 2—figure supplement 1J).

The co-association of eRNA expressing regions with genomic elements associated with active chromatin marks is strongly suggestive of enhancer-like activity. However, to provide further evidence for active ongoing transcription at these loci we performed KAS-seq (Wu et al., 2020) in OE19 cells to identify areas of DNA strand opening as observed in the transcription bubble. The three replicates showed good congruency (Figure 2—figure supplement 2A), and we merged all three to call peaks of DNA strand opening (Supplementary file 4). These peaks show a highly significant overlap with the eRNA regions we identified from patient samples (Figure 2—figure supplement 2B), and higher levels of KAS-seq signal are associated with eRNA regions compared to random regions (Figure 2E). This is exemplified by eRNAs associated with a putative enhancer region located upstream from *JUP* (Figure 2F), *CCNE1* and *MYBL2* gene loci (Figure 2—figure supplement 2C, D). Furthermore, when we combined eRNA regions with regions showing KAS-seq peaks, higher levels of H3K27ac were observed compared to those lacking concomitant KAS-seq signal (Figure 2—figure supplement 2E), indicative of higher activity.

Finally, we focussed on potential super enhancers as these have been shown to play important roles in cancer-specific gene regulation (Hnisz et al., 2013) including gastroesophageal cancers (Ooi et al., 2016). We identified potential super enhancers in OE19 cells using HOMER (Heinz et al., 2010; Whyte et al., 2013) from peak sets generated from H3K27ac ChIP-seq (for histone activation marks) and ATAC-seq (for open chromatin) (Figure 2—figure supplement 3A; Supplementary file 5). We then overlapped these peak sets to generate a dataset with both indicators of super enhancer activity, excluding promoter regions from our analysis. This resulted in 221 high-confidence super enhancers (Figure 2—figure supplement 3B). Next, the constituent enhancers within these super enhancers were overlapped with the eRNA regions we identified from OAC patients, producing a final list of 73 super enhancers showing evidence of eRNA activity, with a total of 216 eRNA regions residing in these super enhancers (Figure 2G). Multiple eRNA regions identified in patient samples are therefore associated with super enhancers as exemplified by the *ELF3* super enhancer (Figure 2—figure supplement 3C). The genes associated with these super enhancers are enriched in several biological processes with direct relevance to OAC, such as MAPK signalling and cadherin binding (Figure 2—figure supplement 3D).

Collectively, these data strongly indicate that the eRNA-associated regions we discovered in patient samples represent areas of enhancer activity due to the presence of enhanced accessibility, enhancer-associated chromatin marks/proteins, and evidence for actively transcribing RNA polymerase in OAC cells.

### Association of eRNA regions to target genes and regulatory transcription factors

**Figure 3.**
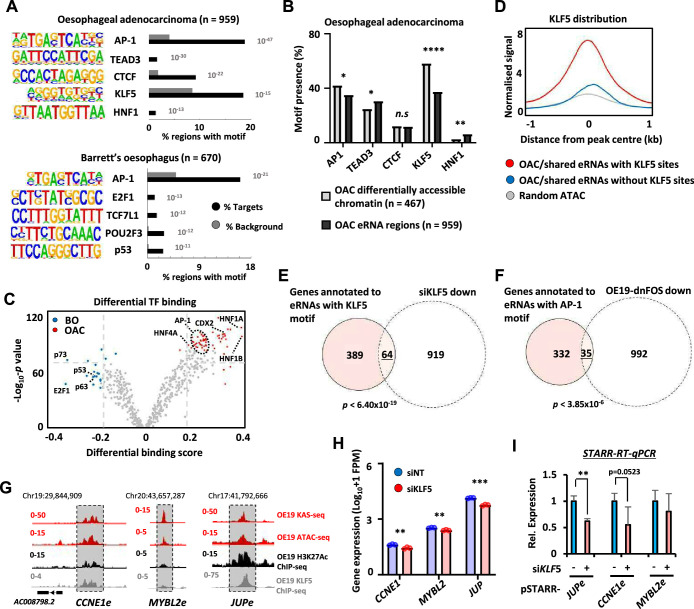
Association of enhancer RNA (eRNA) regions with transcriptional regulators. (**A**) Transcription factor de novo motif enrichment using HOMER, at eRNAs differentially expressed in oesophageal adenocarcinoma (OAC) (top; *n* = 959) and Barrett’s (bottom; *n* = 670) (p values are shown). (**B**) Bar graphs displaying the frequency of motif prevalence of the top 5 enriched motifs at eRNA regions differentially expressed in OAC ( *n* = 959) compared to differentially accessible intergenic chromatin (****p < 0.0001; **p < 0.01; *p < 0.05; *N*-1 Chi-squared test). (**C**) Volcano plot showing differential TF binding (±0.2 differential binding score or ≥−log_10_ p_adj_ 70) at 4600 eRNAs regions using TOBIAS (Bentsen et al., 2020). (**D**) Metaplots of KLF5 ChIP-seq signal from OE19 cells at eRNAs (specific to OAC or shared with Barrett’s oesophagus [BO] eRNA) containing a KLF5 motif, lacking a KLF5 motif or randomly selected open chromatin regions. (**E**) Venn diagram displaying overlap between genes annotated to KLF5 motif containing eRNAs (specific to OAC or shared with BO eRNA) with genes downregulated upon siKLF5 treatment (Log_2_FC ≥1.0, <p_adj_ = 0.05) in OE19 cells (p-value is shown; Fisher’s exact test). (**F**) Venn diagram displaying overlap between genes annotated to AP-1 motif containing eRNAs (specific to OAC or shared with BO eRNA) with genes downregulated upon dominant-negative FOS (dnFOS) induction (Log_2_FC ≥0.5, ≤p_adj_ = 0.05) in OE19 cells (p-value is shown; Fisher’s exact test). (**G**) Genome browser view of KAS-seq, ATAC-seq data, H3K27ac ChIP-seq, and KLF5 ChIP-seq in OE19 cells at the *CCNE1* (left)*, MYBL2* (middle), and *JUP* (right) enhancer loci, with corresponding eRNA regions highlighted (5 kb window shown). (**H**) Bar graphs displaying the expression from RNA-seq analysis of *CCNE1*, *MYBL2*, and *JUP* genes in OE19 cells treated with siRNA targeting KLF5 (*n* = 3; ***p < 0.001; **p < 0.01; Welch’s *t*-test). (**I**) Reverse-transcription quantitative real-time PCR (RT-qPCR) analysis of enhancer activity from the indicated pSTARR-enhancer vectors upon siKLF5 depletion in OE19 cells (*n* = 3; **p < 0.01; *t*-test). See also Figure 3—figure supplement 1.

Next, we asked whether we could identify upstream transcription factors that might control eRNA levels and provide insights into the regulatory landscape of OAC. First, we identified binding motifs for transcription factors that are over-represented in OAC or Barrett’s-specific eRNA producing regions. This revealed enrichment for transcription factors previously identified as relevant for OAC including AP1, KLF5, and HNF1 (Britton et al., 2017; Rogerson et al., 2019; Rogerson et al., 2020; Chen et al., 2020) as well as CTCF, a factor implicated in enhancer activity (Ren et al., 2017; Figure 3A; Supplementary file 6A). However, the frequency of motif occurrence differed between eRNA- and open chromatin-defined enhancers in OAC patients; HNF1 motifs were significantly more enriched in eRNA-defined enhancers whereas AP1 and KLF5 motifs were more enriched in enhancers defined by increased accessibility alone (Figure 3B). A similar set of transcription factor motifs were observed when we omitted regions commonly found in patient samples and OE19 cells and instead focussed on eRNA-defined enhancers inferred only from OAC patient-specific ATAC-seq data (Figure 3—figure supplement 1A). AP1 motifs were again identified in Barrett’s-specific regions as well as a different set of motifs including p53-binding motifs (Figure 3A; Supplementary file 6B). Similarly, calculating differential binding scores revealed higher binding activity of AP1 and HNF1 in OAC-specific regions and conversely higher p53-binding activity in Barrett’s-specific regions (Figure 3C; Supplementary file 7). Thus, eRNA-defined enhancers reveal the activity of disease stage-specific transcriptional regulators. To further explore this point, we sought evidence for enhancer occupancy by transcription factors in OAC cells and found substantially more binding signal of KLF5 derived from ChIP-seq in OE19 cells Rogerson et al., 2020 for eRNA-defined regions with KLF5 motifs compared to regions lacking the motif, or control genomic regions (Figure 3D). Furthermore, evidence for KLF5-mediated regulation was obtained by the significant overlap between the genes associated with the same eRNA regions (ie containing KLF5 motifs) and those genes downregulated upon KLF5 depletion in OE19 cells (Figure 3E; Figure 3—figure supplement 1B–D; Supplementary file 1). We also examined the effect of AP1 inhibition by expressing a dominant-negative FOS derivative (dnFOS; Olive et al., 1997) in OE19 cells (Ogden et al., 2023) and compared the downregulated gene profile with the eRNA-associated genes containing AP1 motifs that we have identified. Again, we observed a significant overlap between the genes associated with eRNA regions containing AP1 motifs in OAC samples and those genes downregulated upon AP1 inhibition (Figure 3F; Figure 3—figure supplement 1E–G; Supplementary file 1). To provide functional links between transcription factor occupancy at enhancers, enhancer activation and target gene transcription, we focussed on KLF5. ChIP-seq data Rogerson et al., 2020 demonstrate that KLF5 strongly binds to the *JUP* and *CCNE1* enhancers but low levels are observed at the *MYBL2* enhancer (Figure 3G). KLF5 depletion (Figure 3—figure supplement 1H) caused reduced expression of all three target genes (Figure 3H). However only *JUP* and *CCNE1* enhancer activities were diminished following KLF5 depletion (Figure 3I), consistent with the higher occupancy of KLF5 at these enhancers compared to *MYBL2. MYBL2* is likely regulated by KLF5 though other cis regulatory elements or by an indirect mechanism. Our data are therefore consistent with KLF5 regulating *JUP* and *CCNE1* expression through the enhancers we have identified. Thus, both motif discovery and functional dissection demonstrate that KLF5 and AP1 are likely major players in eRNA-defined enhancer activation in OAC patients.

**Figure 3—figure supplement 1.**
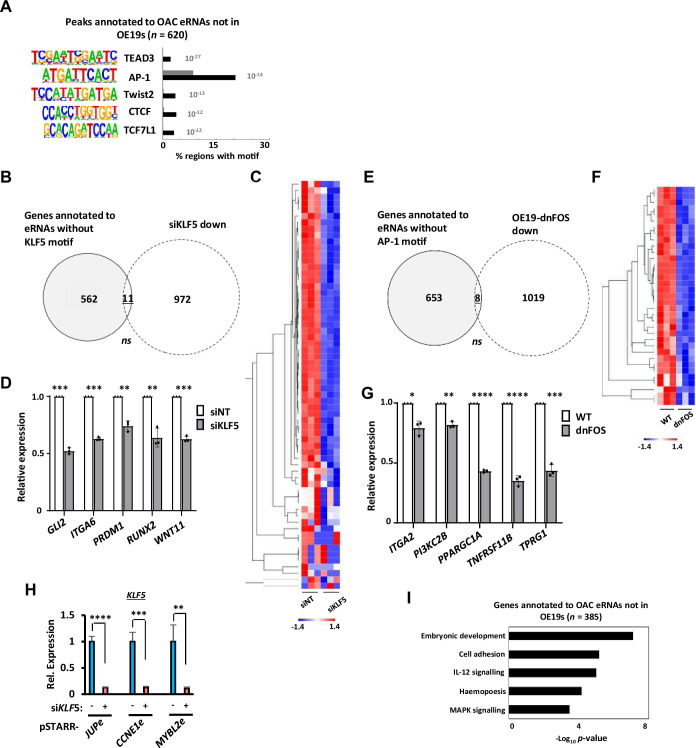
Association of enhancer RNA (eRNA) regions with potential regulatory transcription factors. (**A**) Transcription factor de novo motif enrichment using HOMER, at oesophageal adenocarcinoma (OAC) eRNA containing regions not present in OE19s (*n* = 620; p values are shown). (**B**) Venn diagram displaying overlap between genes annotated to KLF5 motif lacking eRNAs with genes downregulated upon siKLF5 treatment (Log_2_FC ≥1.0,<p_adj_ = 0.05) in OE19 cells (p*-*value is non-significant [ns]; Fisher’s exact test). (**C**) Heatmap and hierarchical clustering of siNT (*n* = 3) and siKLF5 (*n* = 3) RNA-seq samples according to row *z*-score normalised expression of KLF5 eRNA-associated genes which are downregulated upon siKLF5 (*n* = 64). (**D**) Bar graphs displaying difference in expression of five KLF5 eRNA-associated genes which are downregulated upon siKLF5 (*n* = 3; ***p < 0.001; **p < 0.01; Welch’s *t*-test). (**E**) Venn diagram displaying overlap between genes annotated to AP-1 motif lacking eRNAs with genes downregulated upon dominant-negative FOS (dnFOS) induction (Log_2_FC ≥0.5,<p_adj_ = 0.05) in OE19 cells (p-value is non-significant [ns]; Fisher’s exact test). (**F**) Heatmap and hierarchical clustering of wild-type (*n* = 3) and OE19-dnFOS (*n* = 3) RNA-seq samples according to row *z*-score normalised expression of AP-1 eRNA-associated genes which are downregulated upon dnFOS (*n* = 35). (**G**) Bar graphs displaying difference in expression of five AP-1 eRNA-associated genes which are downregulated upon dnFOS (*n* = 3; ****p < 0.0001; ***p < 0.001; **p < 0.01; *p < 0.05; Welch’s *t*-test). (**H**) RT-qPCR analysis of KLF5 expression upon siKLF5 depletion in OE19 cells co-transfected with the indicated pSTARR-enhancer vectors (*n* = 3, ****p < 0.0001; ***p < 0.001; **p < 0.01; *t*-test). (**I**) Gene ontology (GO)-term analysis of genes annotated to OAC eRNAs not present in OE19 cells.

**Figure 4.**
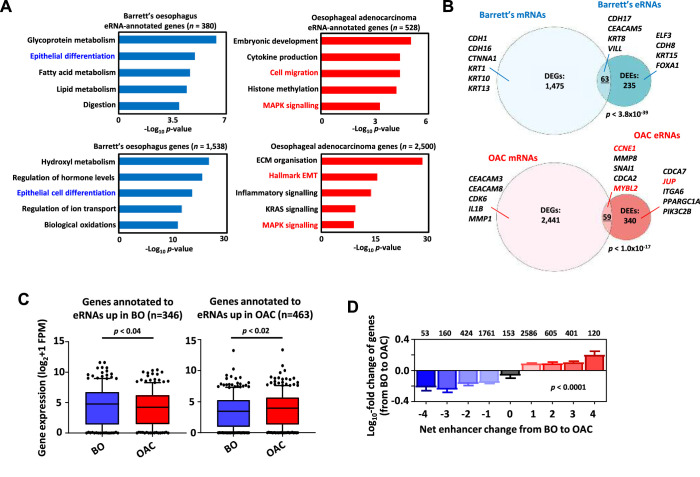
Association of enhancer RNA (eRNA) regions with transcriptional regulators and potential target genes. (**A**) Gene ontology (GO)-term analysis of differentially expressed eRNA region-associated genes (top-left/right) and differentially expressed genes (bottom-left/right) in Barrett’s (left) (>Log_2_FC 0.9, <p_adj_ = 0.05) and oesophageal adenocarcinoma (OAC, right) (>Log_2_FC 1.1, <p_adj_ = 0.05). eRNAs were annotated to genes by the nearest gene model using HOMER (Heinz et al., 2010). (**B**) Venn diagram displaying overlap between differentially expressed genes and unique protein-coding genes annotated to differentially expressed eRNAs in Barrett’s oesophagus (BO, top) and OAC (bottom) (p-value is shown; Fisher’s exact test). (**C**) Box plots comparing the expression of genes annotated to eRNAs differentially expressed in BO (left) or OAC (right) in BO and OAC patient tissue total RNA-seq samples from the OCCAMS dataset (p-value is shown; Welch’s *t*-test). (**D**) Genome-wide analysis of the effect of changing eRNA expression on gene expression within 200 kb chromosomal bins. Numbers above bars represent total genes associated with respective net-enhancer change (p-value is shown; Kruskal–Wallis test). See also Figure 4—figure supplement 1.

To understand the potential biological consequences of eRNA activation, we then linked the differentially active eRNA regions to putative target genes with the nearest gene model using HOMER (Heinz et al., 2010) resulting in 528 genes in OAC and 380 genes in Barrett’s. Gene ontology (GO) analysis identified several terms relevant to the OAC phenotype such as ‘Cell migration’ and ‘MAPK signalling’ whereas Barrett’s-specific regions identified genes associated with various metabolic processes and ‘epithelial differentiation’ that would be expected for this intestinal metaplastic tissue (Figure 4A). A similar set of GO terms were identified when we omitted regions commonly found in patient samples and OE19 cells and instead focussed on eRNA-defined enhancers inferred only from OAC patient-specific ATAC-seq data (Figure 3—figure supplement 1I; Supplementary file 2). One difference was ‘cell adhesion’ which was more enriched in the patient-specific dataset which might reflect the differing adhesive properties on 2D cultured cells and cells growing in a 3D in vivo environment. We also performed differential gene expression analysis on the whole RNA-seq datasets to identify genes preferentially expressed in OAC or Barrett’s and performed GO-term analysis (Supplementary file 8, Supplementary file 9). Similar GO terms were identified with ‘MAPK signalling’ and ‘Hallmark EMT/ECM organisation’ (terms associated with cell migration) resembling those identified through association with eRNA regions (Figure 4A). However, eRNA-associated genes revealed processes not among the top GO terms generated from differentially expressed genes (DEGs) such as ‘embryonic development’ and ‘histone methylation’. Similarly, Barrett’s-specific genes returned GO terms such as ‘epithelial cell differentiation’, as identified from eRNA regions further emphasising the similarity in biological processes identified by eRNA-associated genes and total RNAs. A range of different metabolic processes were also identified in both cases, illustrating the distinct information that is derived from eRNA-associated genes. To determine whether these similar GO categories reflected similar genes being identified, we overlapped the DEGs (Supplementary file 8; Supplementary file 9) with genes associated with differentially expressed eRNAs (DEEsSupplementary file 2; Supplementary file 3), that are enriched in either Barrett’s or OAC samples. We found a significant overlap between these sets of genes although the majority of the genes were uniquely identified by investigating either by total RNA-seq or by eRNA profiling (Figure 4). Therefore, despite pinpointing many similar biological processes, eRNA profiling reveals different candidate genes involved these processes.

**Figure 4—figure supplement 1.**
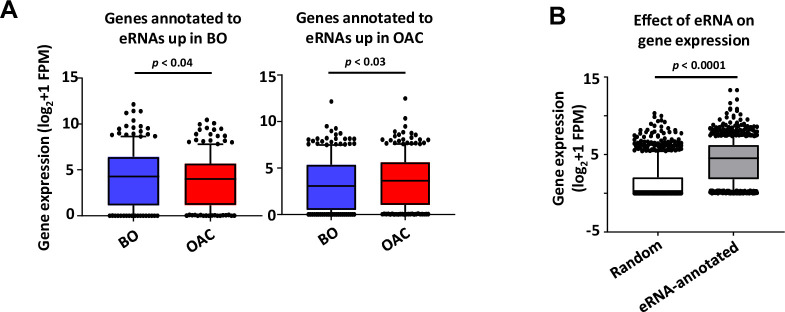
Association of enhancer RNA (eRNA) regions with potential target genes. (**A**) Box plots comparing the expression of genes annotated to eRNAs differentially expressed in Barrett’s oesophagus (BO, left) or oesophageal adenocarcinoma (OAC, right) in BO and OAC patient tissue total RNA-seq samples from the Maag dataset (Maag et al., 2017) (p-value is shown; Welch’s *t*-test). (**B**) Box plots comparing gene expression of 1000 randomly selected genes against genes annotated to eRNAs in the OCCAMs dataset (*n* = 973; p-value is shown; Welch’s *t*-test).

To further investigate whether the activities of enhancer regions are linked to nearby gene expression, we selected eRNA regions preferentially expressed in either OAC or Barrett’s and found that the nearest genes exhibited higher expression in the correct corresponding tissue type in two independent datasets (Figure 4C; Figure 4—figure supplement 1A). This observation was further supported by comparing the expression of the genes closest to eRNA regions found in patient RNA-seq data to the expression of a random set of genes. This revealed significantly enhanced expression levels of eRNA-annotated genes in patients (Figure 4—figure supplement 1B). While the nearest gene model often correctly associates enhancers with the closest gene, this is not always the case, so we considered all genes within a 200 kb bin around the eRNA region rather than just the nearest gene. We then determined the net eRNA expression change when comparing Barrett’s to OAC samples and created nine bins reflecting the magnitude of differential expression. We then calculated the associated gene expression changes within these genomic bins when comparing Barrett’s to OAC. There was a clear correlation between the directionality of eRNA expression with mRNA expression which changed in an analogous manner, with high eRNA levels in OAC associated with higher gene expression in OAC and vice versa in Barrett’s (Figure 4D).

In summary therefore, eRNA expression profiling can reveal specific upstream regulatory transcription factors and the eRNA generating regions can be used to uncover a set of biological processes and constituent genes that are relevant to specific disease states.

### Target genes of eRNA-defined enhancers are co-expressed in OAC

**Figure 5.**
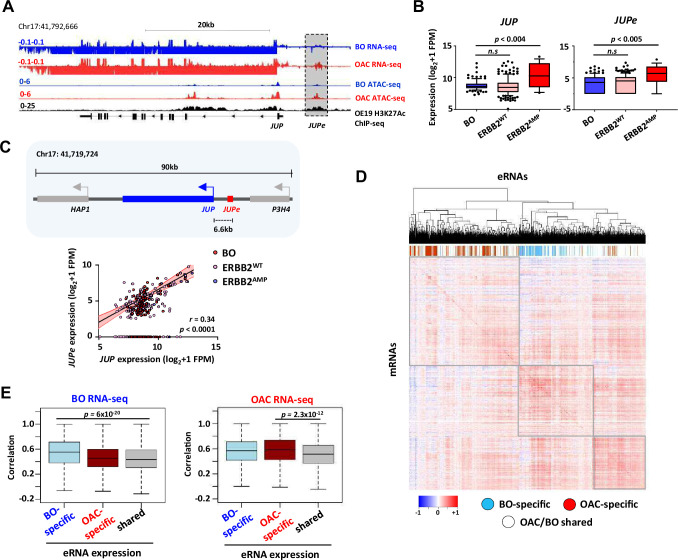
Enhancer RNA (eRNA) regions identify *JUP* as a candidate target gene. (**A**) Genome browser view of Barrett’s oesophagus (BO) and oesophageal adenocarcinoma (OAC) patient tissue ATAC- and total RNA-seq data, and H3K27ac ChIP-seq in OE19 cells, at the *JUP* locus with the *JUPe* eRNA highlighted. (**B**) Box plots comparing the expression of *JUP* (left) and *JUPe* (right) in BO (*n* = 108), ERBB2^WT^ (*n* = 193), and ERBB2^AMP^ (*n* = 17) OAC patient tissue total RNA-seq samples (p-value is shown; Welch’s *t*-test). (**C**) Schematic displaying relative locations of putative eRNA region target genes and nearest neighbours (top) and correlation of *JUPe* with *JUP* expression across BO (*n* = 108), ERBB2^WT^ (*n* = 193), and ERBB2^AMP^ (*n* = 17) OAC patient tissue total RNA-seq samples (bottom) (Spearman’s *r* and p-value are shown; Spearman’s rank correlation test). (**D, E**) Global analysis of correlations of eRNA expression with the expression of the most correlated gene within a 200 kb window flanking the eRNA region. eRNAs are defined as tissue-specific according to Figure 1A, and the rest of the eRNAs are designated as shared. (**D**) Heatmap showing the correlation coefficients between all 4600 eRNAs and the most highly associated mRNAs in the RNA-seq datasets. Samples are clustered based on these correlation coefficients. OAC-specific eRNAs (red), BO-specific eRNAs (blue), and shared eRNAs (white) are indicated across the top. (**E**) Box plots showing the correlations with BO gene expression datasets (left) or OAC gene expression datasets (right). Significance values (*t*-test) are shown between the indicated groups. See also Figure 5—figure supplement 1.

We identified potential target genes of eRNA-defined enhancers by implementing the nearest gene model (Figure 4B). However, the nearest gene is not always the enhancer target (Sanyal et al., 2012). We therefore examined the correlation between eRNA expression and the expression of their designated target genes for three candidate enhancers, localised in the vicinity of the *JUP* (Figure 5A), *MYBL2*, and *CCNE1* (Figure 5—figure supplement 1A, B) loci. Each of these putative enhancer regions contains more RNA signal in OAC compared to Barrett’s as well as evidence for chromatin accessibility in OAC patient material. We focussed on *JUP* as this had not been implicated in OAC previously and is significantly co-amplified with *ERBB2*, a key oncogenic driver of OAC (Cancer Genome Atlas Research Network et al., 2017; Frankell et al., 2019; Figure 5—figure supplement 1C). Indeed, both *JUP* transcript and eRNA are upregulated compared to Barrett’s in OAC patients with high ERBB2 expression (Figure 5B). We performed a similar analysis for *CCNE1* and *MYBL2* but instead examined their expression across all OAC samples. For both loci, the eRNA and gene transcript are both upregulated in OAC relative to Barrett’s (Figure 5—figure supplement 1D, E, ). Next, we examined the correlation of eRNA and transcript expression on a ‘sample by sample’ basis. *JUP* eRNA expression showed strong correlation with *JUP* expression, irrespective of *ERBB2* level sample status (Figure 5C). Lower correlations were observed with the expression of the two adjacent genes (Figure 5—figure supplement 1F) consistent with *JUP* being the relevant target. The correlation between *JUP* eRNA and *JUP-*coding gene expression was even higher when only OAC samples are compared, and this correlation is much lower in Barrett’s samples (Figure 5—figure supplement 1G). Similarly, *CCNE1* and *MYBL2* eRNA expression is more strongly correlated with the expression of their designated targets than either of their immediately flanking genes (Figure 5—figure supplement 1H, I, I). We extended this analysis across all eRNAs and asked whether we could find significantly correlated mRNA expression of genes located in their vicinity (±100 kb)(Supplementary file 10). Generally, highly correlated genes could be identified (see diagonal in Figure 5D). Interestingly when we clustered the data according to the expression of the genes associated with each eRNA, then there was generally a good segregation of OAC- and BO-specific eRNAs further emphasising the relevance of the correlations we observed (Figure 5D). Furthermore, when we split the RNA-seq data into tissue types, there was a significantly higher correlation of BO-specific eRNA with nearby gene expression in BO datasets, compared to analysing the shared eRNAs (Figure 5E, left). Similarly, the same trend was observed for OAC-specific eRNAs which were more highly correlated with nearby gene expression in OAC datasets (Figure 5E, right).

**Figure 5—figure supplement 1.**
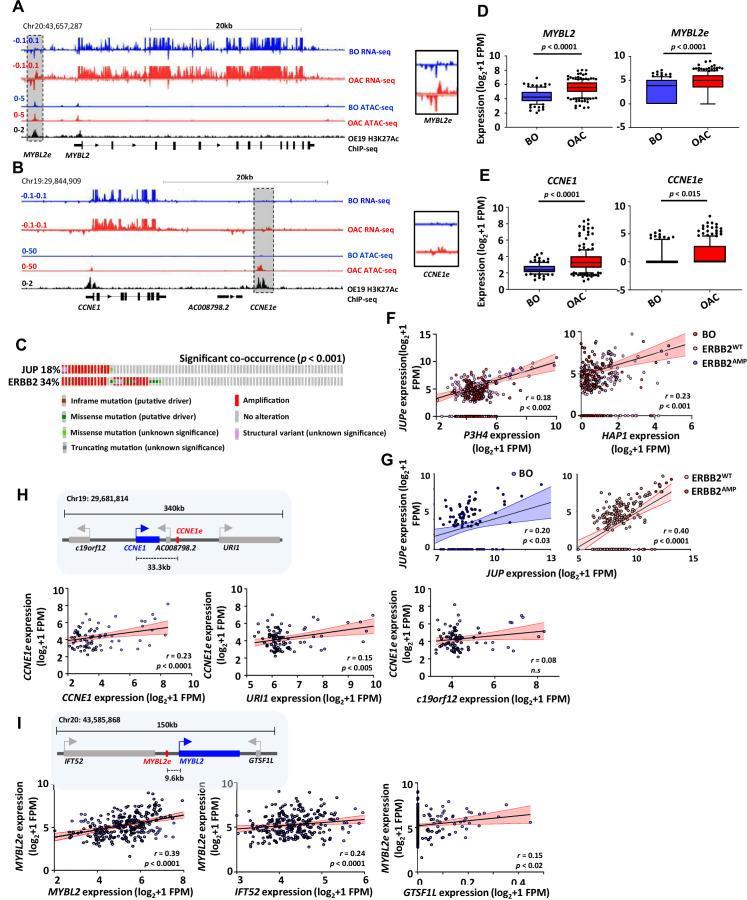
Enhancer RNA (eRNA) regions identify *JUP*, *CCNE1*, and *MYBL2* as a candidate target genes. Genome browser views of Barrett’s oesophagus (BO) and oesophageal adenocarcinoma (OAC) patient tissue ATAC- and total RNA-seq data, and H3K27ac ChIP-seq in OE19 cells, at the *MYBL2* (**A**) and *CCNE1* (**B**) loci with the *MYBL2e and CCNE1e* eRNAs highlighted and inset. (**C**) Oncoprint displaying mutational status of *JUP* and *ERBB2* for OAC patients in the TCGA PanCancer Atlas dataset (p-value is shown; one-sided Fisher’s exact test). Box plots comparing the expression of (**D**) *MYBL2* (left) and *MYBL2e* (right) or (**E**) *CCNE1* (left) and *CCNE1e* (right) in BO (*n* = 108) and OAC (*n* = 210) patient tissue total RNA-seq samples (p-value is shown; Welch’s *t*-test). (**F**) Correlation of *JUPe* and *P3H4* (left) or *HAP1* (right) expression across BO (*n* = 108), ERBB2^WT^ (*n* = 193), and ERBB2^AMP^ (*n* = 17) OAC patient tissue total RNA-seq samples (Spearman’s r and p-value are shown; Spearman’s rank correlation test). (**G**) Correlation of *JUPe* and *JUP* expression in BO (left) or OAC (right) patient total RNA-seq data. Correlation of eRNAs and transcripts for (**H**) *CCNE1e* and *CCNE1* (left)*, URI1* (middle), and *c19orf12* (right) expression and (**I**) *MYBL2e* and *MYBL2* (left)*, IFT52* (middle), and *GTSF1L* (right) expression BO (*n* = 108) and OAC (*n* = 210) patient tissue total RNA-seq samples (Spearman’s *r* and p-value are shown; Spearman’s rank correlation test). Schematics display the relative locations of putative eRNA region target genes and nearest neighbours (top).

Together these results demonstrate that we are able to link eRNA expression to their putative targets in the relevant disease-specific datasets.

### Validation of enhancer activity of eRNA regions

**Figure 6.**
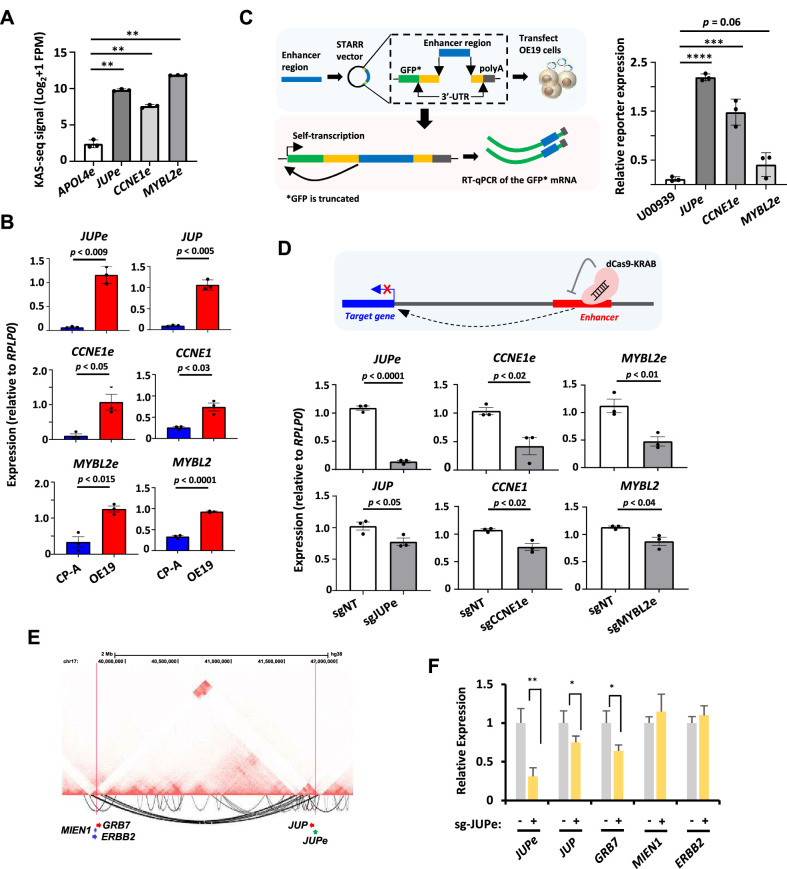
In vitro interrogation of enhancer RNA (eRNA) regions confirms production and association with cancer-associated processes. (**A**) Bar graphs displaying KAS-seq signal at the *APOL4e*, *JUPe*, *CCNE1e*, and *MYBL2e* regions in OE19 cells (*n* = 3; **p < 0.01; Welch’s *t*-test). (**B**) Bar graphs displaying difference in expression of *JUP*, *CCNE1*, *MYBL2* and *JUPe*, *CCNE1e*, and *MYBL2e* between CP-A and OE19 cells using RT-qPCR (*n* = 3; p-value is shown; Welch’s *t*-test; *GFP is truncated). (**C**) Schematic of STARR-RT-qPCR assay (left) and bar graph displaying the difference in STARR reporter activity between *JUPe, CCNE1e*, and *MYBL2e,* compared to *U00930* tRNA-negative control (right) (*n* = 3; ****p < 0.0001; ***p < 0.001; one-way analysis of variance (ANOVA) with Bonferroni’s correction). (**D**) Bar graphs displaying the expression of *JUPe*, *CCNE1e*, and *MYBL2e* eRNAs (top) and *JUP*, *CCNE1*, and *MYBL2* mRNAs (bottom) in OE19-dCas9-KRAB cells using real time RT-qPCR, upon treatment with the indicated targeting or non-targeting (NT) sgRNA (*n* = 3; p-value is shown; Welch’s *t*-test). A schematic of dCas9-KRAB targeting of eRNA regions is shown. (**E**) Genome browser view of Hi-C data surrounding the *JUP* locus. Significant intrachromosomal interactions are shown below the tracks. The start (at *JUP*e) and end (near *ERBB2*) of long range loops are highlighted with red lines. (**F**) RT-qPCR analysis of expression of the indicated genes or *JUP*e eRNAs following dKAS9-KRAB-mediated repression of *JUP*e activity (*n* = 3; **p < 0.01, *p< 0.05; Welch's *t*-test). See also Figure 6—figure supplements 1 and 2.

To validate that the regions generating eRNAs have enhancer activity we again focussed on the *JUP*, *MYBL2*, and *CCNE1* loci. First, we showed that all three putative enhancer regions have significantly higher levels of KAS-seq signal in OE19 cells relative to a control enhancer from the *APOL4* gene that is not expressed in OE19 cells (Figure 6A). This is reflective of ongoing transcription. Furthermore, all three eRNAs and their associated target genes exhibit higher expression in OE19 cells compared to the Barrett’s CP-A cell line (Figure 6B). To directly establish enhancer activity, we cloned the regions encompassing the eRNAs into two different enhancer reporter systems with either RNA (Figure 6C) or luciferase (Figure 6—figure supplement 1A) readouts. For all three regions, both assays demonstrated significant enhancer activity in OE19 cells (Figure 6C and Figure 6—figure supplement 1A). Finally, we used an inducible dCas9-KRAB synthetic repressor protein to silence the activities of each enhancer in their natural chromatin context in OE19 cells (Figure 6—figure supplement 1B). In all cases, introduction of the relevant sgRNA to target the dCas9-KRAB repressor to the putative enhancer, resulted in reduced eRNA transcription and reduced expression of the associated target gene, albeit to a lesser degree in the case of the target genes (Figure 6D). The latter observation is not unexpected as a combination of several proximal and distal regulatory elements rather than a single enhancer likely contributes to their expression. Importantly, no significant changes in expression were observed for any of the genes immediately flanking the target genes, demonstrating the fidelity of our enhancer–gene linkages (Figure 6—figure supplement 1C). However, to establish whether any additional longer range enhancer–gene linkages could be found, we used Hi-C to generate a 3D chromatin map in OE19 cells. Replicate samples showed good reproducibility (stratum-adjusted correlation coefficient = 0.965). However, we were unable to identify any long range interactions emanating from the *CCNE1*e and *MYBL2*e enhancer regions (Figure 6—figure supplement 2A, B). In contrast, we identified significant long range linkages between the *JUP*e enhancer and the region located downstream from the *ERBB2* locus (Figure 6E). This juxtapositioning likely arises due to genomic rearrangements that are seen in OAC where JUP is often located on the same ecDNA amplicons as *ERBB2* (Ng et al., 2022). We tested whether any of the genes in the vicinity of the contact point are also regulated by the JUPe region and found that *GRB7* expression is reduced upon reducing JUPe activity, but no effect is seen on *ERBB2* or *MIEN1* expression (Figure 6F). *GRB7* expression is also reduced following KLF5 depletion, consistent with the role of KLF5 in activating this enhancer region (Figure 6—figure supplement 2C).

**Figure 6—figure supplement 1.**
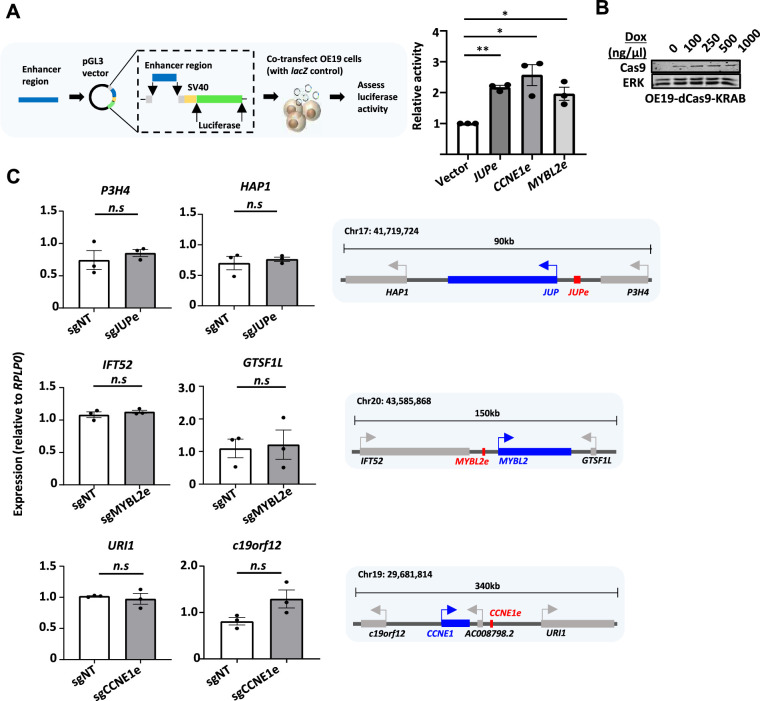
In vitro interrogation of enhancer RNA (eRNA) regions confirms production and association with cancer-associated processes. (**A**) Schematic of luciferase assay (left) and bar graph displaying the luciferase reporter activity between *JUPe*, *CCNE1e*, and *MYBL2e,* compared relative to vector only negative control (right) (*n* = 3; **p < 0.01; *p < 0.05; one-way analysis of variance (ANOVA) with Bonferroni’s correction). (**B**) Western blot showing induction of Cas9 in OE19-dCas9-KRAB cells upon doxycycline treatment. (**C**) Bar graphs displaying difference in expression of (top) *P3H4* and *HAP1* upon sgJUPe treatment*,* (middle) *IFT52* and *GTSF1L* upon sgMYBL2e treatment, and (bottom) *URI1* and *c19orf12* upon sgCCN1E treatment, in OE19-dCas9-KRAB cells using RT-qPCR (*n* = 3; p-value is shown; Welch’s *t*-test). A diagrammatic representation of the genomic region surrounding each enhancer is shown on the right. Figure 6—figure supplement 1—source data 1.Raw unedited images of Western blots.Membranes have been probed for ERK1/2 as a loading control and Cas9. The regions used for creating the final figure are boxed. Molecular weight marker sizes (kDa) are shown on the right. Figure 6—figure supplement 1—source data 2.Original TIFF files used to create Figure 8, Figure 6—figure supplement 1b.

**Figure 6—figure supplement 2.**
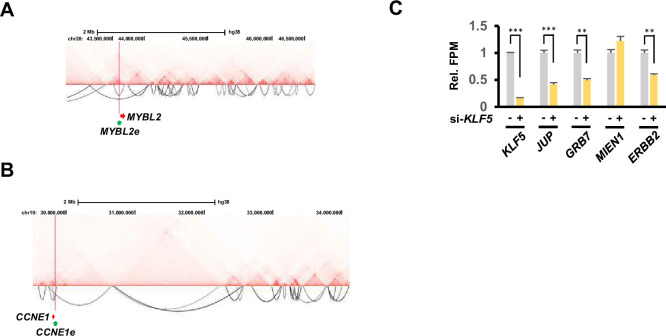
HiC analysis of OE19 cells. Genome browser views of Hi-C data surrounding the *MYBL2* (**A**) and *CCNE1* (**B**) loci (genes depicted as red arrows). Significant intrachromosomal interactions are shown below the tracks. The positions of the *MYBL2e-* and *CCNE1e-*defined enhancers are indicated. (**C**) RNA-seq analysis of expression of the indicated genes or *JUP*e enhancer RNAs (eRNAs) following siRNA-mediated depletion of KLF5 (*n* = 3; ***p< 0.001, **p < 0.01; *t*-test).

Together these results build on our correlative observations linking eRNA containing regions with enhancer-like properties and provide definitive proof of enhancer activity and regulatory linkage to neighbouring genes.

### Biological and clinical relevance of eRNAs and their target genes

**Figure 7.**
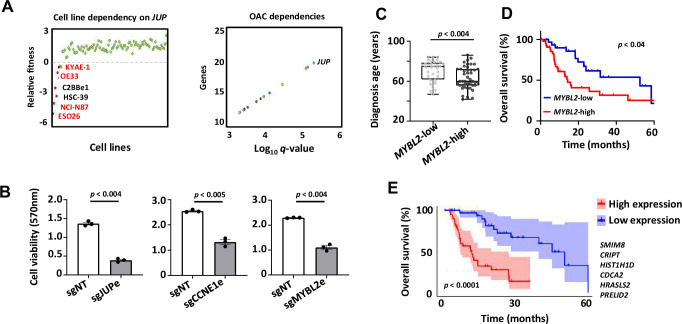
Biological and clinical relevance of enhancer RNAs (eRNAs) and their target genes. (**A**) Scatter plots displaying data from the Sanger DepMap Project Score (Tsherniak et al., 2017; Behan et al., 2019) highlighting cell line dependency on *JUP* (left) (gastroesophageal cell lines are marked in red) and top genetic dependencies in oesophageal adenocarcinoma (OAC, right). (**B**) Bar graph displaying the difference in cell viability in OE19-dCas9-KRAB cells upon sgRNA treatment, assessed by crystal violet assay (*n* = 3; p-value is shown; Welch’s *t*-test). (**C**) Box plots comparing diagnosis age for OAC patients with low and high *MYBL2* expression in the TCGA PanCancer Atlas dataset (p-value is shown; Welch’s *t*-test). (**D**) Kaplan–Meier plot comparing overall survival between OAC patients with low and high *MYBL2* expression in the TCGA PanCancer Atlas dataset (Log rank p-value is shown). (**E**) Kaplan–Meier plot comparing overall survival between OAC patients with low and high signature eRNA target expression in the TCGA PanCancer Atlas dataset (Log rank p-value is shown; signature genes are shown). See also Figure 7—figure supplement 1.

We have shown that the discovery of eRNAs in OAC patients reveals genes and processes which are operative in OAC and allows us to distinguish OAC from Barrett’s patients. To provide further biological insights, we asked whether any of the three eRNA target genes, *JUP*, *MYBL2*, and *CCNE1* were uncovered in a cell line viability screen in the DepMap project (Tsherniak et al., 2017; Behan et al., 2019). We found that four of the top 6 cell lines showing a dependency on *JUP* expression are gastroesophageal in origin and these all contain *ERBB2* amplifications (Figure 7A, left). *JUP* is also the highest scoring gene for fitness dependency across OAC cell lines (Figure 7A, right). In contrast, *GRB7* which is also JUPe-regulated does not majorly contribute to the fitness of OAC cell lines (Figure 7—figure supplement 1A). Similarly, *MYBL2* and *CCNE1* did not score highly in this screen. We therefore further probed the function of the eRNA-defined enhancers in the OE19 OAC cell line by using the dCas9-KRAB silencing system directed at these regions. In all cases, enhancer silencing led to significant reductions in cell viability and growth (Figure 7B; Figure 7—figure supplement 1B).

**Figure 7—figure supplement 1.**
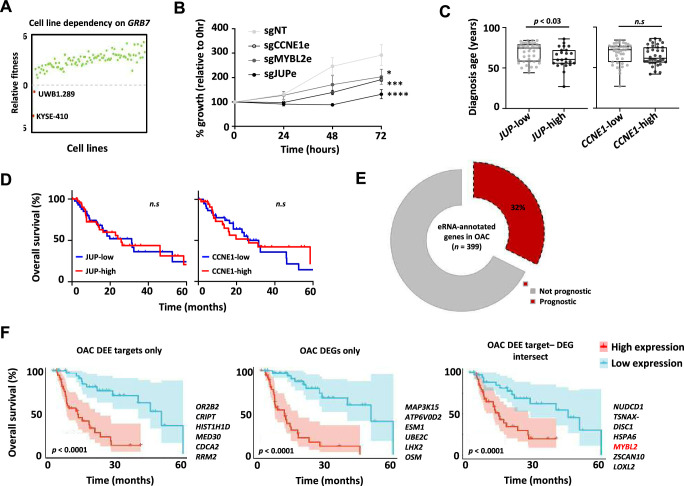
Biological and clinical relevance of enhancer RNAs (eRNAs) and their target genes. (**A**) Scatter plots displaying data from the Sanger DepMap Project Score (Tsherniak et al., 2017; Behan et al., 2019) highlighting cell line dependency on *GRB7*. KYSE-410 is squamous oesophageal and UWB1.289 is ovarian cancer in origin. (**B**) Growth curves comparing difference in growth in OE19-dCas9-KRAB cells upon indicated sgRNA treatment, assessed by crystal violet assay (*p < 0.05, ***p < 0.001, ****p < 0.0001; two-way analysis of variance [ANOVA]). (**C**) Box plots comparing diagnosis age for oesophageal adenocarcinoma (OAC) patients with low and high *JUP* (left) and *CCNE1* (right) in the TCGA PanCancer Atlas dataset (p-value is shown; Welch’s *t*-test). (**D**) Kaplan–Meier plots comparing overall survival between OAC patients with low and high *JUP* expression (left) and *CCNE1* expression (right) in the TCGA PanCancer Atlas dataset (Log rank p-value is shown). (**E**) Number of OAC eRNA-annotated genes that are prognostic for patient survival in the TCGA PanCancer Atlas dataset (Log rank p-value <0.05). (**F**) Kaplan–Meier plots comparing overall survival between OAC patients with low and high expression of six-gene signatures drawn from the genesets in each sector of Figure 4B; OAC DEE target genes (left), OAC-specific differentially expressed genes (DEGs, middle), or OAC DEE target/OAC DEG intersect (right) in the TCGA PanCancer Atlas dataset (log rank p-value and signature genes are shown).

To provide further clinical relevance, we used an RNA-seq dataset from the TCGA consortium (Cancer Genome Atlas Research Network et al., 2017) that differed from our discovery cohort to ask whether any of the eRNA target genes informed on any particular clinical features. We found that the age of diagnosis was lower in patients expressing high levels of *MYBL2* (Figure 7C) and *JUP* (Figure 7—figure supplement 1C) suggesting earlier disease onset. Furthermore, in the case of *MYBL2*, high-level expression was indicative of lower median survival times (Figure 7D), although *JUP* and *CCNE1* were not informative in that regard (Figure 7—figure supplement 1D). For *JUP*, this is not unexpected as it is only amplified in a subset of patients where co-amplification with *ERBB2* is often observed (Figure 7—figure supplement 1C). Altogether, 32% of genes annotated to OAC-specific eRNAs displayed a significant prognostic value for patient survival (Figure 7—figure supplement 1E). Finally, we took an unbiased approach and asked whether we could identify a clinically significant signature within the entire eRNA-associated gene list. This revealed a six-gene signature that was highly predictive of OAC patient survival (Figure 7E). Only three out of six genes comprising this signature were annotated to OAC DEEs. We therefore also explored whether we could derive a prognostic signature from OAC unique DEE-annotated genes, OAC unique DEGs or the intersect of OAC DEEs and DEGs (as defined in Figure 4B) and identified differing prognostic signatures in all categories (Figure 7—figure supplement 1F). DEEs are therefore able to predict prognostic signatures on their own to an equivalent level as using DEGs but do so by providing alternative molecular markers to assess patient prognosis.

Collectively, these data demonstrate the functional importance of the eRNA-defined enhancers and their target genes for OAC cell growth and their potential utility for assessing patient prognosis. In the case of *JUP*, the broad OAC cell dependency suggests that this represents a target of potential therapeutic value, especially in *ERBB2*-positive patients.

## Discussion

Cancer is driven by a combination of genetic and epigenetic changes (reviewed in Zhao et al., 2021). Both of these processes ultimately lead to alterations in the activity of gene regulatory elements, including transcriptional enhancers, that results in a change in cellular phenotype that defines the tumourigenic state. While profiling of histone marks and chromatin accessibility is useful in defining potential gene regulatory elements, this approach is limited for defining active enhancers. Here, we used eRNA profiling to identify regions harbouring potentially active enhancers in OAC patient samples. We integrated these with a range of epigenetic datasets and experimentally validated several regions as *bona fide* enhancers. Importantly, our enhancer repertoire identified new molecular events that are activated in OAC which were not apparent from either genome sequencing or mRNA profiling alone.

A previous pan-cancer analysis of RNA-seq datasets generated by the TCGA consortium to identify eRNAs defined a compendium of potential enhancers across human cancers and demonstrated how they could have clinical significance (Chen et al., 2018). However, while the authors examined oesophageal cancers, they mixed two distinct disease sub-types, squamous and adenocarcinoma, which limited any discoveries specific to OAC. Here, we specifically interrogated OAC RNA-seq data (generated by the OCCAMs consortium) and to identify enhancers that are potentially relevant to OAC, we compared their associated eRNA levels to the pre-cancerous BO state. Using this approach, we were able to identify ~1000 high-confidence OAC-specific enhancers. These enhancer regions exhibited high accessibility in both patient samples and cell line models and using a variety of chromatin marks profiled in an OAC cell line model we provided further verification of the enhancer-like properties. The OAC-specific enhancers are associated with transcription factors which have been shown to be important for driving OAC-specific transcriptional events (e.g., KLF5, Rogerson et al., 2020; AP1, Britton et al., 2017). Reciprocally we also identify ~700 Barrett’s-specific enhancers which are associated with a different transcription factor repertoire, including the potential involvement of members of the TP53/TP63/TP73 family. While we have identified a large number of intergenic enhancers, the approach we have taken will miss intragenic enhancers, and other approaches using function-based assays (e.g., STARR-seq; Arnold et al., 2013) or computational imputation will be needed to identify these.

Our newly derived eRNA-defined enhancer datasets also provide novel insights into pathways that are operational in OAC. This is apparent from the limited overlap in DEGs at the mRNA level versus the differential expression of genes associated with nearby enhancers defined by eRNA levels. Interestingly while the specific gene overlaps are limited, the broad processes defined by GO terms such as MAPK signalling and cell migration/EMT remain the same. Part of this discrepancy might be explained by the OAC-specific enhancers maintaining gene expression in the BO–OAC transition rather than de novo gene activation in OAC or alternatively that many genes may be primarily driven by changes in promoter rather than enhancer activity. However, expression change cut offs we use may also contribute to this, as can the heterogeneity of the OAC samples. Other potential reasons for the lack of congruency include technical issues such as the nearest gene model may not always provide the most appropriate linkage of eRNAs to target genes and that we are likely missing many eRNAs due to the datasets we used which are not optimally designed for eRNA identification. An enhancer associated with *JUP* was specifically revealed by eRNA profiling, alongside hundreds of other enhancers linked to genes involved in oncogenic processes such as cell migration, PI3K signalling, and metabolism. We validated this *JUP* enhancer, and enhancers linked to *MYBL2* and *CCNE1*, and their association with their proposed targets by CRISPRi. Furthermore, correlations between eRNA and mRNA expression across cancer samples suggest a causal link. Longer range regulatory interactions were detected between the *JUP* enhancer and *GRB7*, but HiC analysis did not reveal any additional potential connections for the other two enhancers. It remains possible that such long regulatory interactions may exist that escaped detection using HiC. Importantly both CCNE1 and MYBL2 play important roles in promoting cell proliferation, an important cancer cell trait. JUP (otherwise known as junction plakoglobin) had not previously been implicated in OAC but this was identified in a screen for gene dependencies in OAC cell lines (DepMap project: Tsherniak et al., 2017; Behan et al., 2019) and we validated its importance for OAC cell growth. In this context, the co-amplification with ERBB2 is intriguing as both genes are on the same chromosome and are ~2 Mb apart and the intervening region is not usually co-amplified. Both can be found on the same ecDNA molecules (Ng et al., 2022) allowing closer juxtapositioning of *ERBB2* and the *JUPe* enhancer, which we were able to validate using HiC in OE19 cells. Nevertheless, we were unable to detect *JUPe* enhancer-mediated ERBB2 regulation. This co-amplification may instead reflect a functional interdependency for these two oncogenic events. JUP has previously been implicated in multiple cancers although it is generally found to be a tumour suppressor protein, rather than the oncogenic properties it has in the context of OAC (reviewed in Aktary et al., 2017). As JUP encodes a protein involved in cell–cell contacts, this might suggest a role for this process in OAC cancer cell survival and a potential route to therapy. Alternatively, JUP may be acting via the numerous other cellular processes in which it has been implicated, and further work is needed to understand the precise role it has in OAC cells.

In addition to pointing to potential actionable pathways, we also demonstrate that eRNA profiling is clinically relevant and is sufficient to differentiate between BO and OAC. A six-gene signature derived from our OAC-specific enhancer-associated genes is able to predict prognostic outcomes. Indeed, a large proportion of the eRNA-associated genes show prognostic significance when analysed on an individual basis. Furthermore, by focussing in on a few examples, we found that one of the novel OAC-associated genes, *JUP*, was upregulated in *ERBB2* overexpressing OAC samples which is reflected by their frequent co-amplification. Coupled with the observation that JUP is required for the survival of a range of OAC cell lines harbouring *ERBB2* amplifications, this further emphasises the potential utility of JUP as a therapeutic target in this subset of OAC patients. This would provide an alternative approach to the use of ERBB2 inhibitors which are routinely administered but have limited therapeutic benefit (Bang et al., 2010). Further clinical insights are provided by other eRNA-defined enhancer regions, such as the enhancer associated with *MYBL2* where high *MYBL2* expression indicates a worse prognosis for patients and earlier disease onset.

In summary, we identify a cohort of OAC-specific enhancers, expanding our knowledge of the regulatory networks that are operational in OAC. This has led to novel insights into the pathways that are operational in this disease. The approach we have taken to identify cancer-specific enhancers should be broadly applicable to other tumour types or subtypes, where data are available for both the cancer and the originating normal or pre-cancerous tissue.

## Materials and methods

### Cell culture and treatments

OE19 cells were purchased from ATCC and tested negative for mycoplasma. OE19 cells were cultured in RPMI 1640 (Thermo Fisher Scientific, 52400) supplemented with 10% foetal bovine serum (Thermo Fisher Scientific, 10270). OE19-dCas9-KRAB stable cells were previously generated from the parental OE19 cells (Rogerson et al., 2020) and cultured as above with the addition of 500 ng/ml puromycin (Sigma P7255). The expression of dCas9-KRAB was induced using 100 or 250 ng/ml doxycycline (Sigma-Aldrich, D3447). Cell lines were cultured at 37°C, 5% CO_2_ in a humidified incubator.

### dnFOS over-expression

pINDUCER20-GFP-AFOS (ADS5006, Britton et al., 2017) was packaged into lentivirus and OE19 cells were transduced with lentivirus as previously described (Tiscornia et al., 2006). Briefly, 3 × 10^6^ HEK293T cells were transfected with 2.25 μg psPAX2 (Addgene, 12260), 1.5 μg pMD2.G (Addgene, 12259), and 3 μg pINDUCER20-GFP-AFOS using PolyFect (Qiagen, 301107). Media was collected at 48 and 72 hr post-transfection and viral particles were precipitated using PEG-it Solution (System Biosciences, LV810A-1). To transduce, cells were treated with virus (Multiplicity of Infection (MOI) 0.5–1.0) and 5 μg/ml Polybrene (EMD Millipore, TR-1003). Polyclonal cells were selected for 2 weeks in 250 μg/ml G418 (Thermo Fisher Scientific, 10131027). dnFOS (Olive et al., 1997) was induced with 1 μg/ml doxycycline.

### sgRNA transfection

2 × 10^5^ cells were transfected with 10 pmol sgRNA pool using Lipofectamine RNAiMAX transfection reagent (Thermo Fisher Scientific, 13778150) according to the manufacturer’s instructions. Cells were seeded into 6-well plates. Modified full-length sgRNAs were designed using E-CRISP (Heigwer et al., 2014) and off-target activity assessed using CCTop (Stemmer et al., 2015). sgRNAs were ordered from Synthego. sgRNA sequences are listed in Supplementary file 11.

### Cell growth and cell viability assays

Cell growth and viability were assessed by crystal violet assay. Assays were performed by fixing cells in 4% paraformaldehyde for 10 min. Cells were stained with 0.1% crystal violet (Sigma-Aldrich, HT90132) for 30 min. Crystal violet dye was extracted using 10% acetic acid and absorbance readings taken at 570 nm on a SPECTROstar Nano Microplate Reader (BMG LABTECH). Cell growth measurements were taken at 0, 24, 48, and 72 hr and cell viability measurements taken at 72 hr.

### RT-qPCR and eRNA qPCR

Total RNA was extracted from cells using an RNeasy Plus RNA extraction kit (Qiagen, 74136) according to the manufacturer’s protocol. RT-qPCR reactions were run using the QuantiTect SYBR Green RT-qPCR kit (Qiagen, 204243) on a Qiagen Rotor-Gene Q. For eRNA-qPCR, RNA was extracted using an RNeasy Plus RNA extraction kit (Qiagen, 74136) with the on-column DNAse digest, according to the manufacturer’s instructions. 500 ng of RNA was reverse-transcribed using SuperScript VILO Master Mix (Thermo Fisher Scientific, 11755250) according to the manufacturer’s instructions. eRNA levels were assessed by qPCR using a Rotor-Gene SYBR Green PCR Kit (Qiagen, 1054586) on a Qiagen Rotor-Gene Q. Relative transcript levels were determined by standard curve and normalised to the expression of *RPLP0* control gene. Primers used are listed in Supplementary file 11.

### Luciferase and STARR-qPCR reporter assays

Regions containing JUPe, MYBL2e, or CCNE1e were amplified from OE19 genomic DNA using primers containing 20 bp overlap regions with the multiple cloning site of the pGL3 Promoter vector (Promega, E1761) for luciferase assays, or between the InFusion arms of the hSTARR_ORI vector (Addgene, 99296) (Supplementary file 11). Final vectors were assembled using HiFi assembly (NEB, E5520S) according to the manufacturer’s instructions to create plasmids containing JUPe, MYBL2e, or CCNE1e enhancer regions in either hSTARR_ORI (pAS5008-pAS5010) or pGL3- vectors (pAS5011-pAS5013). All recombinant plasmids are available upon request. Enhancer vectors were transfected using the Amaxa Nucleofector II (Lonza) with Cell Line NucleofectorTM Kit V (Lonza, VCA-1003) and program T-020, according to the manufacturer’s instructions. For luciferase assays, 250 ng of enhancer vector was co-transfected alongside 50 ng of pCH110 (Amersham). For STARR-qPCR, 800 ng of vector was transfected. Enhancer activity was assessed using the Dual-Light Luciferase & β-Galactosidase Reporter System (Thermo Fisher Scientific, T1003) according to the manufacturer’s instructions, or by RT-qPCR.

### Western blots

Cells were lysed in Radioimmunoprecipitation assay (RIPA) buffer (1% IGEPAL CA-630, 150 mM NaCl, 0.1% sodium dodecyl sulfate [SDS], 50 mM Tris pH 8.0, 1 mM Ethylenediaminetetraacetic acid (EDTA), 0.5% sodium deoxycholate) and protease inhibitor cocktail supplement (Roche, 11836170001). Protein concentration was determined by bicinchoninic acid assay (Pierce, 23227). 5× SDS loading buffer (235 mM SDS, 10% β-mercaptoethanol, 0.005% bromophenol blue, 210 mM Tris–HCl pH 6.8, 50% glycerol) was added to lysates to a final 1× concentration and incubated for 10 min at 90°C. Proteins were then resolved by SDS–polyacrylamide gel electrophoresis and transferred onto a nitrocellulose membrane. Membranes were blocked using Odyssey Blocking Buffer (LI-COR Biosciences, P/N 927-40000). Antibodies used: anti-Cas9 (Diagenode, C15200229, 1:10,000) and anti-ERK (Cell Signaling Technologies, 4695S, 1:1000). Secondary antibodies used: anti-rabbit (LI-COR Biosciences, 926-32213, 1:10,000) and anti-mouse (LI-COR Biosciences, 926-32210, 1:10,000). Membranes were visualised using a LI-COR Odyssey CLx Infrared Imager.

### eRNA and mRNA analysis

Patient tissue ATAC-seq data processing was performed as described previously (Britton et al., 2017). Reads were mapped to GRCh38 (hg38) using Bowtie2 v2.3.0 (Langmead and Salzberg, 2012) with the following options: -X 2000 -dovetail. Mapped reads ( ≥ q30) were retained using SAMtools (Li et al., 2009). Reads mapping to blacklisted regions were removed using BEDtools (Quinlan and Hall, 2010). Peaks were called using MACS2 v2.1.1 (Zhang et al., 2008) with the following parameters: -q 0.01, -nomodel-shift –75 -extsize 150 -B –SPMR. A custom union peakset was formed from all BO and OAC patient samples, using HOMER v4.9 mergePeaks.pl -d 250 (Heinz et al., 2010) as described previously (Rogerson et al., 2019) and filtered to retain only intergenic regions ≥2 kb upstream from a TSS or ≥500 bp downstream from a TTS.

RNA-seq reads were mapped to the human genome GRCh38 (hg38) using STAR v2.3.0 (Dobin et al., 2013). The expression threshold for eRNAs was determined using an adapted method from Zhang et al., 2019. Briefly, total RNA-seq reads were integrated into genomic regions from the intergenic patient ATAC-seq peakset. Putative eRNA and mRNA read counts were determined using featureCounts (Liao et al., 2014) and FPM values determined using DESeq2 (Love et al., 2014). Putative eRNA regions with average counts and FPM values of ≥3 and 1.5, respectively, were taken forward for further analysis. Differentially expressed eRNAs and mRNAs were determined using DESeq2 (Love et al., 2014). For eRNAs, a log_2_-fold change of ±0.5 and p-value_adj_ <0.05 defined differential expression. For BO and OAC mRNAs, a log_2_-fold change of ±0.9 and ±1.5, respectively, and p-value_adj_ <0.05 defined differential expression. ERBB2-positive OAC samples (*ERBB2*^AMP^) were determined based on these samples having expression of *ERBB2* greater than the median *ERBB2* expression +2 SD. Morpheus (https://software.broadinstitute.org/morpheus/) was used to generate heatmaps and perform hierarchical clustering.

HOMER v4.9 was used for de novo transcription factor motif enrichment analysis. To analyse footprinting signatures at putative eRNA regions, TOBIAS v0.5.1 was used (Bentsen et al., 2020). eRNAs were annotated to genes by the nearest gene model and assessed for CpG content using HOMER v4.9. Super enhancers were identified using HOMER v4.9 findPeaks.pl -style super. Net enhancer activity was calculated as in Bi et al., 2020. Briefly, neighbouring genes of eRNA regions in both BO and OAC were identified and stratified into nine groups based on the net eRNA change within 200 kb of the TSS of each gene: + (or −1) stands for 1 net gained (or lost) eRNA from BO to OAC. Bidirectionality score was calculated using HOMER v4.9 analyzeRepeats.pl with the −strand option applied for each strand and score defined as log_10_((+strand expression score + 1)/(−strand expression score + 1)) + 1.

### DepMap data

Batch-corrected genome-wide CRISPR–Cas9 knockout screen data (DepMap Public 21Q4 CRISPR_gene_dependency.csv) were obtained from DepMap (https://depmap.org/portal/).

### ChIP-seq data analysis

ChIP-seq analysis was carried out as described previously (Wiseman et al., 2015). OE19 H3K27ac and GAC H3K4me1/3 ChIP-seq reads were mapped to the human genome GRCh38 (hg38) using Bowtie2 v2.3.0 (Langmead and Salzberg, 2012). Biological replicates were checked for concordance (*r* > 0.80). Peaks were called using MACS2 v2.1.1, using input DNA as control (Zhang et al., 2008). Mapped reads (≥q30) were retained using SAMtools (Li et al., 2009). Reads mapping to blacklisted regions were removed using BEDtools (Quinlan and Hall, 2010).

### CUT&Tag processing and data analysis

CUT&Tag library generation was performed as described previously (Kaya-Okur et al., 2020) with an altered nuclear extraction step. For the nuclear extraction, OE19 cells were initially lysed in Nuclei EZ lysis buffer (Sigma-Aldrich, NUC-101) at 4°C for 10 min followed by centrifugation at 500 × *g* for 5 min. The subsequent clean-up was performed in a buffer composed of 10 mM Tris–HCl pH 8.0, 10 mM NaCl and 0.2% NP40 followed by centrifugation at 1300 × *g* for 5 min. Nuclei were then lightly cross-linked in 0.1% formaldehyde for 2 min followed by quenching with 75 mM glycine followed by centrifugation at 500 × *g* for 5 min. Cross-linked nuclei were resuspended in 20 mM N-2-hydroxyethylpiperazine-N'-2-ethanesulfonic acid (HEPES) pH 7.5, 150 mM NaCl, and 0.5 M spermidine at a concentration of 4–8 × 10^3^ / μl (2–4 × 10^4^ total). Subsequent stages were as previously described (Kaya-Okur et al., 2020). For 2–4 × 10^4^ nuclei, 0.5 μg of primary and secondary antibodies were used with 1 μl of pA-Tn5 (Epicypher, 15-1017). Antibodies used: anti-BRD4 (abcam, ab128874), anti-CTCF (Merck-Millipore, 07-729), anti-H3K27ac (abcam, ab4729), anti-H3K27me3 (Merck-Millipore, 07-449), anti-H3K4me1 (abcam, ab8895), anti-H3K4me2 (Diagenode, pAb-035-010), anti-H3K4me3 (abcam, ab8580), anti-H3K36me3 (Diagenode, pAb-058-010), anti-H4K20me1 (Diagenode, mAb-147-010), anti-PolII (abcam, ab817), anti-PolII-S2 (abcam, ab5095), anti-PolII-S5 (abcam, ab5131), and anti-Med1 (AntibodyOnline, A98044/10 UG). CUT&Tag libraries were pooled and sequenced on an Illumina HiSeq 4000 System (University of Manchester Genomic Technologies Core Facility). CUT&Tag data processing was performed as for ChIP-seq but with the MACS2 v2.1.1 (Zhang et al., 2008) but the --broad peak calling option was used for the H4K20me1, H3K27me3 and H3K36me3 marks. Fraction reads in peak (FRiP) scores for each mark were calculated using featureCounts and a stringent threshold of ≥2% was set to ensure quality of data for downstream analyses (Landt et al., 2012; FRiP scores are listed in Supplementary file 12).

ChromHMM (Ernst and Kellis, 2012) was used to train an eight-state HMM using the CUT&Tag data for all marks assayed. The number of states was determined by running the model with increasing numbers of states until state separation was observed. Emission states were annotated in accordance with Roadmap Epigenomics Consortium Data (Kundaje et al., 2015).

### KAS-seq processing and data analysis

KAS-seq library generation was performed as described previously (Wu et al., 2020) except with nuclear extraction and labelling reactions. Nuclei were extracted and washed as described for CUT&Tag. Nuclei were then resuspended in nuclease-free H_2_O at a concentration of 1 × 10^4^/μl (2 × 10^5^ total). Labelling reactions were carried out in DNA LoBind tubes (Eppendorf, 0030108051) using 5 mM N_3_-kethoxal (a gift from Chuan He) in phosphate-buffered saline to a final volume of 50 μl for 15 min at 37°C with 1000 RPM mixing in a thermomixer. Labelled gDNA was isolated using the PureLink Genomic DNA Mini kit (Thermo Fisher Scientific, K182001) and eluted twice with 21.5 μl 25 mM K_3_BO_3_ pH 7.0. Subsequent library preparation stages were as previously described (Wu et al., 2020). KAS-seq libraries were pooled and sequenced on an Illumina HiSeq 4000 System (University of Manchester Genomic Technologies Core Facility). Three biological replicates were sequenced and checked for concordance (*r* > 0.80). KAS-seq data processing was performed as described previously (Wu et al., 2020), but with the MACS2 v2.1.1 --broad peak calling option.

### HiC analysis

HiC samples for mammalian cells were carried out using the Arima-HiC Kit (A510008, ARIMA Genomics) with some modifications. Briefly, the nuclei were prepared from 3 million cross-linked cells (−80°C) using Nuclei EZ prep (NUC101, Sigma-Aldrich) at 4°C for 10 min and spun down 500 × *g* at 1°C for 5 min. The nuclei wash was carried out in 0.09% bovine serum albumin (BSA)/CapC lysis buffer (10 mM Tris–Cl pH 8.0, 10 mM NaCl, 0.2% NP40, 0.09% BSA, and 1 tablet of EDTA-free protease inhibitor cocktail (11873580001, Roche) per 50 ml) at 4°C for 10 min and spun down at 500 × *g* at 1°C for 5 min. The nuclei pellets were resuspended in 25 μl of nuclease-free H_2_O (total volume of nuclei is ~30 μl). A 20-μl solution (~2 million) of freshly prepared nuclei was used for HiC sample preparation.

HiC libraries were generated using the Arima Library Prep module (A303011, ARIMA Genomics) as described by the manufacturers and sequenced using a NovaSeq6000 (Illumina). We used Illumina 150 bp paired end sequencing (300 cycle) to obtain ~1 billion read-pairs per sample.

The HiC dataset consists of the two biological replicated samples in OE19 cells. The paired-end reads of each sample were aligned to the human genome hg38 by the aligning software BWA-MEM v0.7.17 (Li and Durbin, 2010). The uniquely mapped reads were processed by the HiC data analysis pipeline Juicer v1.6 (Durand et al., 2016). The contacts identified in each of the two samples were stored in the.hic files. We applied the R package HiCRep with the default settings (Yang et al., 2017) to the contacts at MAPQ ≥ 30 to calculate the stratum-adjusted correlation coefficient (SCC) between the two replicates. As HiCRep calculated the SCC for the contacts on each chromosome, we calculated the chromosome-length weighted average of the SCCs on all the chromosomes as a summary SCC. The summary SCC for the two replicates is 0.965. We also applied the Juicer pipeline to the pool of the aligned reads from the two replicates and obtained the contacts from the merged reads of the two replicates.

The HiC data files of the two samples were uploaded in ArrayExpress repository with the ArrayExpress data ID E-MTAB-12664.

### The Cancer Genome Atlas data

Diagnosis age and overall survival between OAC patients with high or low *JUP*, *CCNE1*, and *MYBL2* RNA expression (defined as ±1 SD from the median expression) in the TCGA PanCancer Atlas dataset (Liu et al., 2018) were downloaded from cBioPortal (https://www.cbioportal.org/study/summary?id=esca_tcga_pan_can_atlas_2018). Oncoprint plot of mutational co-occurrence between *JUP* and *ERBB2* in OAC was generated using cBioPortal.

To establish the prognostic model, univariate Cox regression was performed using the survival package in R v3.6.0 to select genes associated with patient prognosis utilising a criteria of *q*-value <0.1. A random forest algorithm was applied using the randomForestSRC package in R v3.6.0 for feature reduction to obtain a survival signature. Risk score (risk score = ∑x_*i*_ × *β*_*i*_ where x_*i*_ is gene expression value; *β*_*i*_ is coefficient index) was calculated using a multivariate Cox regression model. Patients were grouped by the median value of risk score and Kaplan–Meier analysis performed to compare the survival difference between high- and low-risk score group. Visualisation was achieved using the survminer package in R v3.6.0.

### Bioinformatics

Genome browser data were visualised using the UCSC Genome Browser (Kent et al., 2002). Heatmaps and tag density plots of epigenomic data were generated the using deepTools (Ramírez et al., 2016) computeMatrix, plotProfile, plotCorrelation, and plotHeatmap functions. Metascape (Zhou et al., 2019) was used for GO analysis of gene sets. The eulerr package in R v3.6.0 was used for generating Venn diagrams.

### Datasets

All data were obtained from ArrayExpress, unless stated otherwise. Human tissue RNA-seq data were obtained from: OCCAMS consortium (European Genome-Phenome Archive, EGAD00001007496). Human tissue ATAC-seq data were obtained from: E-MTAB-5169 (Britton et al., 2017), E-MTAB-6751 (Rogerson et al., 2019), and E-MTAB-8447 (Rogerson et al., 2020). The Cancer Genome Atlas OAC ATAC-seq data were obtained from the GDC data portal (https://portal.gdc.cancer.gov/; Corces et al., 2018). OE19 H3K27ac ChIP-seq was obtained from: E-MTAB-10319 (Ogden et al., 2022). GAC H3K4me1 and H3K4me3 ChIP-seq were obtained from: Gene Expression Omnibus, GSE75898 (Ooi et al., 2016). OE19 siKLF5 RNA-seq and KLF5 ChIP-seq were obtained from: E-MTAB-8446 and E-MTAB-8568, respectively (Rogerson et al., 2020). OE19 dnFOS RNA-seq was obtained from E-MTAB-10334 (Ogden et al., 2023).

### Data access

All data have been deposited at ArrayExpress; OE19 KAS-seq and CUT&Tag data (E-MTAB-11357 and E-MTAB-11356, respectively), and OE19 HiC data (E-MTAB-12664).

## Data Availability

All data have been deposited at ArrayExpress; OE19 KAS-seq and CUT&TAG data (E-MTAB-11357 and E-MTAB-11356, respectively) and OE19 HiC data (E-MTAB-12664). The following datasets were generated: AhmedI YangSH OgdenS ZhangW LiY SharrocksAD OCCAMS Consortium 2022KAS-seq in OE19 cellsArrayExpressE-MTAB-11357 AhmedI YangSH OgdenS ZhangW LiY SharrocksAD OCCAMS Consortium 2022CUT&TAG of OE19 cell lineArrayExpressE-MTAB-11356 AhmedI YangSH OgdenS ZhangW LiY SharrocksAD OCCAMS Consortium 2023HiC data in OE19 cellsArrayExpressE-MTAB-12664 The following previously published datasets were used: OoiWF XingM XuC YaoX RamleeMK LimMC CaoF LimK BabuD PoonLF Lin SulingJ QamraA IrwantoA Qu ZhengzhongJ NandiT Lee-LimAP ChanYS TayST LeeMH DaviesJO WongWK SooKC ChanWH OngHS ChowP WongCY RhaSY LiuJ HillmerAM HughesJR RozenS TehBT FullwoodMJ LiS TanP 2016Somatic Promoter Landscape of Primary Gastric Adenocarcinoma Delineated by Epigenomic ProfilingNCBI Gene Expression OmnibusGSE75898 OgdenS CarysK BruceJ The OCCAMS Consortium SharrocksAD 2021Sequencing data for oesophageal and related samples - Ogden et al releaseEuropean Genome Phenome ArchiveEGAD00001007496 BrittonE RogersonC MehtaS LiY LiX FitzgeraldRC AngYS SharrocksAD 2017ATAC-seq of oesophageal cell lines and tissue samplesArrayExpressE-MTAB-5169 RogersonC BrittonE WitheyS HanleyN AngYS SharrocksAD 2019ATAC-seq of human Barrett's oesophagus tissueArrayExpressE-MTAB-6751 RogersonC BrittonE WitheyS HanleyN AngYS SharrocksAD 2020ATAC-seq of oesophageal adenocarcinoma patient samplesArrayExpressE-MTAB-8447 RogersonC OgdenS BrittonE OCCAMS Consortium AngYS SharrocksAD 2020RNA-seq of OE19 cells treated with siNT or siKLF5 for 72 hoursArrayExpressE-MTAB-8446 RogersonC OgdenS BrittonE OCCAMS Consortium AngYS SharrocksAD 2020KLF5 ChIP-seq in CP-A and OE19 cellsArrayExpressE-MTAB-856810.7554/eLife.57189PMC754450432880368 OgdenS AhmedI YangS-H FullwoodP The OCCAMS Consortium FrancavillaC SharrocksAD 2023OE19 dnFOS RNA-seqArrayExpressE-MTAB-1033410.1093/narcan/zcad001PMC986907836694726 OgdenS CarysK BruceJ The OCCAMS Consortium SharrocksAD 2021ChIP-seq of H3K27Ac in oesophageal adenocarcinoma OE19 cellsArrayExpressE-MTAB-10319 CorcesMR GranjaJM ShamsS LouieBH SeoaneJA ZhouW SilvaTC GroeneveldC WongCK ChoSW SatpathyAT MumbachMR HoadleyKA RobertsonAG SheffieldNC FelauI CastroMAA BermanBP StaudtLM ZenklusenJC LairdPW CurtisC Cancer Genome Atlas Analysis Network GreenleafWJ ChangHY 2018TCGA-generated ATAC-seq of oesophageal adenocarcinoma patient samplesNIH GDCATAC-AWG

## Additional files

Supplementary file 1. All expressed eRNAs identified in Barrett’s and OAC samples.

Supplementary file 2. eRNAs upregulated in OAC.

Supplementary file 3. eRNAs upregulated in Barrett’s.

Supplementary file 4. KAS-seq peaks in OE19 cells.

Supplementary file 5. Super enhancers.

Supplementary file 6. Motif enrichment.

Supplementary file 7. BINDetect scores.

Supplementary file 8. Genes upregulated in OAC.

Supplementary file 9. Genes upregulated in Barrett’s.

Supplementary file 10. Genes associated with eRNA regions.

Supplementary file 11. PCR primer and sgRNA sequences.

Supplementary file 12. CUT&Tag FRIP scores.

MDAR checklist

## Appendix 1

**Appendix 1—key resources table:**

Reagent type (species) or resource	Designation	Source or reference	Identifiers	Additional information
Cell line (*H. sapiens*)	OE19	ACACC	96071721	
Cell line (*H. sapiens*)	CP-A	ATCC	KR-42421	
Cell line (*H. sapiens*)	OE19-dCas9-KRAB	Rogerson et al., 2020		OE19 transfected with vector to express dCas9-KRAB under doxycycline control
Antibody	Rabbit monoclonal anti-Erk1/2 antibody	Cell Signalling Technology	4695S	(1:1000)
Antibody	Donkey polyclonal anti-mouse secondary antibody (800CW)	Licor	925–32,212	(1:10,000)
Antibody	Donkey polyclonal anti-rabbit secondary antibody (700CW)	Licor	925–32,213	(1:10,000)
Antibody	Mouse monoclonal anti-Cas9	Diagenode	C15200229	(1:10,000)
Antibody	Rabbit monoclonal anti-BRD4	Abcam	ab128874	0.5 µg/2–4 × 10^5^ cells
Antibody	Rabbit polyclonal anti-CTCF	Merck-Millipore	07-729	0.5 µg/2–4 × 10^5^ cells
Antibody	Rabbit polyclonal anti-H3K27ac	Abcam	ab4729	0.5 µg/2–4 × 10^5^ cells
Antibody	Mouse polyclonal anti-H3K27me3	Merck-Millipore	07-449	0.5 µg/2–4 × 10^5^ cells
Antibody	Rabbit polyclonal anti-H3K4me1	Abcam	ab8895	0.5 µg/2–4 × 10^5^ cells
Antibody	Rabbit polyclonal anti-H3K4me2	Diagenode	pAb-035-010	0.5 µg/2–4 × 10^5^ cells
Antibody	Rabbit poylclonal anti-H3K4me3	Abcam	ab8580	0.5 µg/2–4 × 10^5^ cells
Antibody	Rabbit poylclonal anti-H3K36me3	Diagenode	pAb-058-010	0.5 µg/2–4 × 10^5^ cells
Antibody	Mouse monoclonal anti-H4K20me1	Diagenode	mAb-147-010	0.5 µg/2–4 × 10^5^ cells
Antibody	Mouse monoclonal anti-PolII	Abcam	ab817	0.5 µg/2–4 × 10^5^ cells
Antibody	Rabbit polyclonal anti-PolII-S2	Abcam	ab5095	0.5 µg/2–4 × 10^5^ cells
Antibody	Rabbit polyclonal anti-PolII-S5	Abcam	ab5131	0.5 µg/2–4 × 10^5^ cells
Antibody	Rabbit polyclonal anti-Med1	https://www.antibodies.com/	A98044/10 UG	0.5 µg/2–4 × 10^5^ cells
Recombinant DNA reagent	pGL3 reporter vector	Promega	E1761	
Recombinant DNA reagent	hSTARR_ORI vector	Addgene	99296	
Recombinant DNA reagent	pINDUCER20-GFP-AFOS	Britton et al., 2017		ADS5006
Recombinant DNA reagent	pCH110	Amersham		
Recombinant DNA reagent	pMD2.G	Addgene	12259	
Recombinant DNA reagent	psPAX2	Addgene	12260	
Sequence-based reagent	Primers	This study		Primers for amplification through PCR (See supplementary file). Primers can be ordered through any commercial vendor.
Commercial assay or kit	Lipofectamine RNAiMAX	Thermo Fisher	13778150	
Commercial assay or kit	Cell Line NucleofectorTM Kit V	Lonza	VCA-1003	Used on Amaxa Nucleofector II with program T-020
Commercial assay or kit	Dual-Light Luciferase & β-Galactosidase Reporter System	Thermo Fisher	T1003	
Commercial assay or kit	SuperScript VILO Master Mix	Thermo Fisher	11755250	
Commercial assay or kit	PureLink Genomic DNA Mini kit	Thermo Fisher	K182001	
Commercial assay or kit	HiFi assemly	NEB	E5520S	
Commercial assay or kit	QuantiTect SYBR Green RT-PCR Kit	Qiagen	204243	
Commercial assay or kit	RNeasy Plus Mini Kit	Qiagen	74134	
Commercial assay or kit	RNase-free DNase set	Qiagen	79254	
Commercial assay or kit	Ampure XP beads	Beckman Coulter Agencourt	A63881	
Commercial assay or kit	TruSeq stranded RNA library kit v2	Illumina	RS-122-2001	
Commercial assay or kit	Nextera DNA library prep kit	Illumina	FC-121-1031	
Commercial assay or kit	Nextera Index kit	Illumina	FC-121-1012	
Commercial assay or kit	NEBNext high fidelity 2x PCR master mix	NEB	M0541	
Commercial assay or kit	DNA Clean and Concentrator	Zymo	D4013	
Commercial assay or kit	Polyfect	Qiagen	301107	
Commercial assay or kit	PEG-it	System Biosciences	LV810A-1	
Commercial assay or kit	Polybrene	EMD Millipore	TR-1003	
Chemical compound, drug	Doxycycline	Sigma-Aldrich	D3447	Used at final concentration of 100 ng/ml
Chemical compound, drug	N_3_-kethoxal	Gift from Chuan He		Used at 5 mM
Peptide, recombinant protein	RNase	Sigma	R4642	Used at 100 μg/ml
Peptide, recombinant protein	pA-Tn5	Epicypher	15-1017	
Peptide, recombinant protein	EGF	Thermo Fisher	10450-013	5 μg/l
Peptide, recombinant protein	Bovine pituitary extract	Thermo Fisher	1E+07	Used at 50 mg/l
Software, algorithm	Trimmomatic	Bolger et al., 2014	V0.34	http://www.usadellab.org/cms/?page=trimmomatic
Software, algorithm	ChromHMM	Ernst and Kellis, 2012		
Software, algorithm	Bowtie2	Langmead and Salzberg, 2012	v2.3.0	http://bowtie-bio.sourceforge.net/bowtie2/index.shtml
Software, algorithm	STAR	Dobin et al., 2013	V2.5.4	https://github.com/alexdobin/STAR
Software, algorithm	Macs2	Zhang et al., 2008	v2.1.1	https://github.com/taoliu/MACS
Software, algorithm	DEseq2	Love et al., 2014	V1.22.2	https://bioconductor.org/packages/release/bioc/html/DESeq2.html
Software, algorithm	TOBIAS	Bentsen et al., 2020	v0.5.1	https://github.com/loosolab/TOBIAS
Software, algorithm	featureCounts	Liao et al., 2014	V1.6.2	http://subread.sourceforge.net
Software, algorithm	FastQC		v0.11.4	https://www.bioinformatics.babraham.ac.uk/projects/fastqc/
Software, algorithm	bedtools	Quinlan and Hall, 2010	v2.26.0	https://bedtools.readthedocs.io/en/latest/
Software, algorithm	DeepTools	Ramírez et al., 2016	V2.5.0	https://deeptools.readthedocs.io/en/develop/
Software, algorithm	Metascape	Zhou et al., 2019		https://metascape.org/gp/index.html
Software, algorithm	Homer	Heinz et al., 2010	v4.9	http://homer.ucsd.edu/homer/
Software, algorithm	R	R Development Core Team, 2018	v3.5.1	https://www.r-project.org/
Software, algorithm	GraphPad Prism		V8.0	https://www.graphpad.com/
Software, algorithm	Morpheus	Broad Institute		https://software.broadinstitute.org/morpheus/
Other	Crystal violet	Sigma-Aldrich	HT90132	Histological DNA stain. Used at concentration of 0.1%
Other	Gibco RPMI 1640	Thermo Fisher	52400	Cell culture medium for OE19s.
Other	Gibco fetal bovine serum	Thermo Fisher	10270	Cell culture supplement.
Other	Gibco penicillin/streptomycin	Thermo Fisher	15140122	Cell culture supplement
Other	Keratinocyte SFM (1×)	Thermo Fisher	17005042	Cell culture medium for CP-As.
