# Supplementary material for: eRNA profiling uncovers the enhancer landscape of oesophageal adenocarcinoma and reveals new deregulated pathways
Source: eLife. 2023 Feb 20;12:e80840. doi: 10.7554/eLife.80840 (PMC9998086; doi:10.7554/eLife.80840)
Supplement: Figure 6—figure supplement 1—source data 1. — Membranes have been probed for ERK1/2 as a loading control and Cas9. The regions used for creating the final figure are boxed. Molecular weight marker sizes (kDa) are shown on the right. [file elife-80840-fig6-figsupp1-data1.zip › Figure 6- figure supplement 1B- source data/Figure 6 - figure supplement 1B-source data1.pptx]

## Slide 1
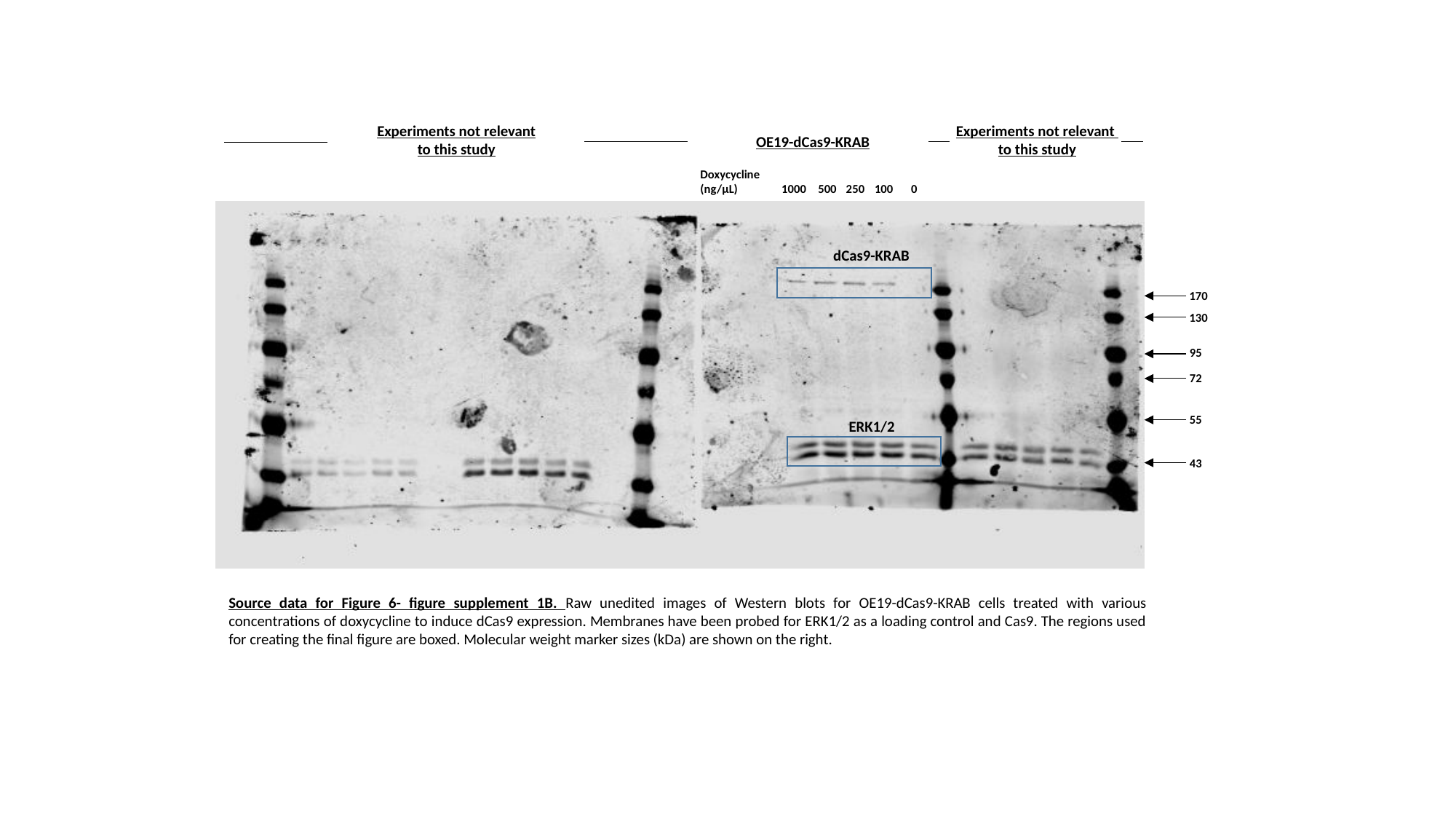

Experiments not relevant
to this study
Experiments not relevant
to this study
OE19-dCas9-KRAB
Doxycycline (ng/μL)
1000
500
250
100
0
dCas9-KRAB
170
130
95
72
55
ERK1/2
43
Source data for Figure 6- figure supplement 1B. Raw unedited images of Western blots for OE19-dCas9-KRAB cells treated with various concentrations of doxycycline to induce dCas9 expression. Membranes have been probed for ERK1/2 as a loading control and Cas9. The regions used for creating the final figure are boxed. Molecular weight marker sizes (kDa) are shown on the right.
