# Supplementary figures and images for: eRNA profiling uncovers the enhancer landscape of oesophageal adenocarcinoma and reveals new deregulated pathways
Source: eLife. 2023 Feb 20;12:e80840. doi: 10.7554/eLife.80840 (PMC9998086; doi:10.7554/eLife.80840)

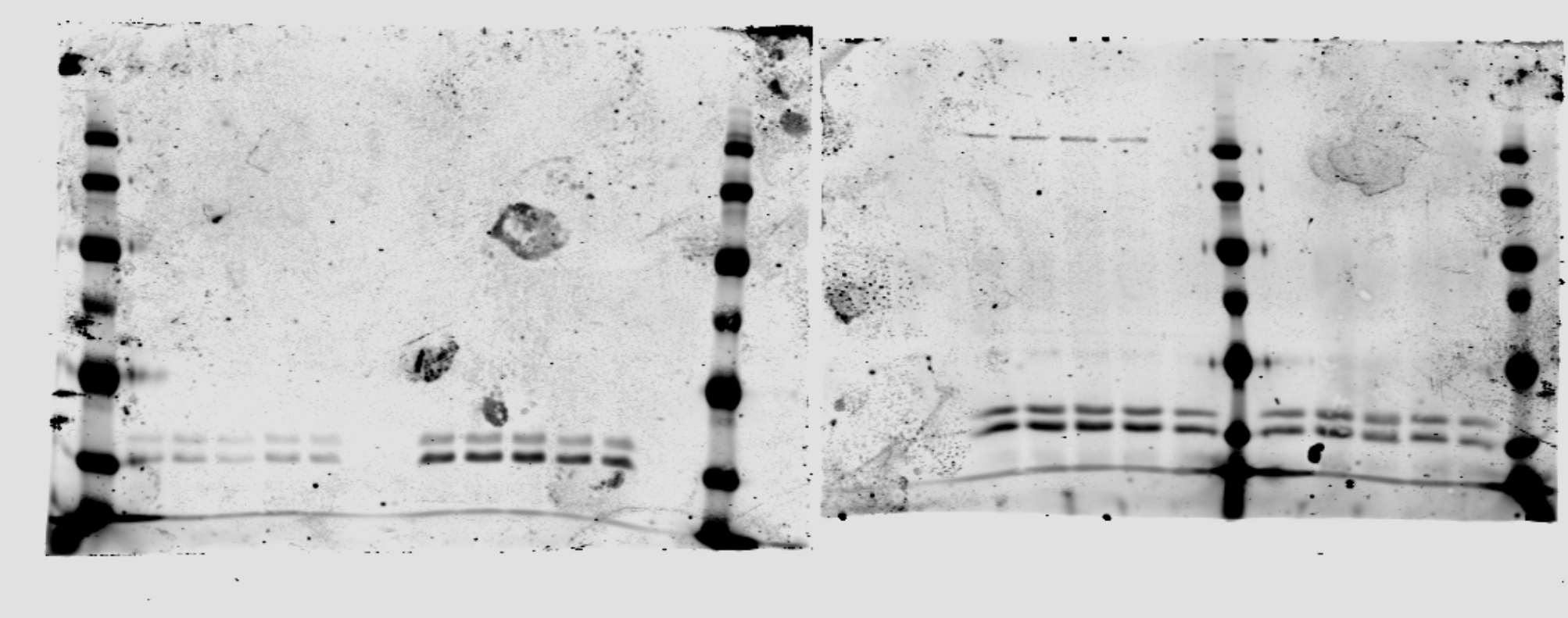

Supplement: Figure 6—figure supplement 1—source data 1. — Membranes have been probed for ERK1/2 as a loading control and Cas9. The regions used for creating the final figure are boxed. Molecular weight marker sizes (kDa) are shown on the right. [file elife-80840-fig6-figsupp1-data1.zip › Figure 6- figure supplement 1B- source data/Figure 6 - figure supplement 1B-source data2.tif]
