# Supplementary material for: eRNA profiling uncovers the enhancer landscape of oesophageal adenocarcinoma and reveals new deregulated pathways
Source: eLife. 2023 Feb 20;12:e80840. doi: 10.7554/eLife.80840 (PMC9998086; doi:10.7554/eLife.80840)
Supplement: Peer review history

# Editor report: Editor's evaluation

**DOI:** 10.7554/eLife.80840.sa0

**Authors:** William C Hahn

**Affiliations:** https://ror.org/02jzgtq86 Dana-Farber Cancer Institute United States

This is an important study that identifies enhancer-associated (e)RNAs specifically associated with Oesophageal adenocarcinoma (OAC). Integrative analyses of patient gene expression data and epigenetic data from an OAC-derived cell line provide convincing support for the identification of eRNAs as markers of enhancers that are important for OAC tumour biology. This paper should be of wide general interest to researchers interested in how epigenetics drive cancer development.

# Decision letter

**DOI:** 10.7554/eLife.80840.sa1

**Affiliations:** https://ror.org/02jzgtq86 Dana-Farber Cancer Institute United States; https://ror.org/0015ws592 Institut de Génétique et de Biologie Moléculaire et Cellulaire France

Our editorial process produces two outputs: (i) public reviews designed to be posted alongside the preprint for the benefit of readers; (ii) feedback on the manuscript for the authors, including requests for revisions, shown below. We also include an acceptance summary that explains what the editors found interesting or important about the work.

**Decision letter after peer review:**

Thank you for submitting your article "eRNA profiling uncovers the enhancer landscape of oesophageal adenocarcinoma and reveals new deregulated pathways" for consideration by *eLife*. Your article has been reviewed by 3 peer reviewers, and the evaluation has been overseen by a Reviewing Editor and Richard White as the Senior Editor. The following individual involved in the review of your submission has agreed to reveal their identity: Irwin Davidson (Reviewer #3).

The reviewers have discussed their reviews with one another, and the Reviewing Editor has drafted this to help you prepare a revised submission.

Essential revisions:

1) The authors discuss AP1, KLF5, and HNF1 in Figure 3. However, they have not demonstrated that eRNAs specific to OACs also relate to their ability to transcribe target genes. They should consider if these TFs regulate JUP based as a function of JUPe expression. As one example, upon CRISPRi of JUPe, they should determine that these factors have diminished regulatory activity of JUP transcripts through ChIP-qPCR coupled with the existing RT-PCR experiments.

2) Enhancers may regulate transcript of more than one gene. The authors should consolidate the relationship between eRNA expression of JUP, CCNE1, and MYBL2, based on structural interactions. A potential experiment is Hi-C sequencing.

3) From the text, it is not clear how the authors defined the 6-gene signature that was used to associate with OS in Figure 7E this should be better described (there is a description in the Methods section, but it could be made clearer). Also, can the authors demonstrate that a signature based on differentially expressed eRNA-associated genes has better clinical value than signatures based on differentially expressed genes? As mentioned above the data in Figure 4 shows only limited overlap. For example, do the 59 overlapping AOC genes in Figure 4B allow better association with the clinical outcome than signatures based on the most differentially expressed genes or the 340 most differentially expressed eRNA-associated genes? Is the 6-gene signature in Figure 7E present in this list?

4) Some of the data in Figure 5 is not very convincing. For example, the enhancer activity measured by the pGL3 vectors is very weak, whereas it is much more convincing with the STARR vectors. The pGL3 data could be removed. Also, can the authors express the data in 5D as fold change over the control vector rather than 'reporter expression'. Similarly, the changes in JUP, CCNE1, and MYBL2 in panel E are rather weak compared to the changes in the corresponding eRNAs. Given the extent of these changes, it is difficult to understand the strong effects on viability in Figure 7B. Can the authors comment?

5) There is a significant amount of new CUT&TAG data, and it seems that this could have been more clearly presented. For example, although Figure 2C shows a correlation between different marks, this figure does not show how these marks correlate with eRNA regions compared to randomly accessible regions. Figure S2C shows this, but the comparison is not quantitative, and not all marks are included.

6) The authors should put the context of the percentages of eRNAs found at enhancers and other regions to make it clear which findings are significant.

*Reviewer #1 (Recommendations for the authors):*

1. The authors discuss AP1, KLF5, and HNF1 in Figure 3. However, they have not demonstrated that eRNAs specific to OACs also relate to their ability to transcribe target genes. They should consider if these TFs regulate JUP based as a function of JUPe expression. As one example, upon CRISPRi of JUPe, they should determine that these factors have diminished regulatory activity of JUP transcripts through ChIP-qPCR coupled with the existing RT-PCR experiments.

2. Enhancers may regulate the transcript of more than one gene. The authors should consolidate the relationship between eRNA expression of JUP, CCNE1, and MYBL2, based on structural interactions. A potential experiment is Hi-C sequencing.

3. Based on the same concerns, in Figure 6E – the transcript expression suppressing effects of targeting the eRNA is small when examining JUP, CCNE1, and MYBL2, despite robust suppression of the eRNAs. However, this also corresponds to a significant viability effect in Figure 7B. The concern is that the eRNA represents a measure of regulatory activity on more than one target gene and that other targets also contribute to the viability regulating phenotypes. The authors should demonstrate the specificity of the experiments. As one example, related to JUPe, authors should examine other genes on 17q, such as ERBB2. A key demonstration would be that these 17q genes are not associated with JUPe in OAC samples, that JUPe suppression does not impact their expression in cells, and that suppression of these other 17q genes does not have viability effects in the same set of cell lines. Based on Hi-C experiments, they should also confirm there is no interaction with ERBB2 or other genes.

4. Outside of JUPe, the authors have not examined if JUP has general roles in OACs without ERBB2 amplifications. In Figure 7A, the cell line results from Depmap include 6 ERBB2 amplified models. In Figure S7C there are no OACs with JUP amplification outside of samples with ERBB2 amplification. This coincides with the lack of OS in Figure S9C.

5. A concern is that JUPe measurements are a surrogate for cell activity that is not specific to JUP expression. In Figure 5C, JUPe is not expressed in many samples that have clear JUP expression, which suggests JUPe is not essential for JUP expression. This figure panel is also hard to resolve since BO and OAC samples are in the same plot. The authors should consider splitting Figure 5C into two panels to demonstrate that JUP expression is tightly regulated by JUPe in OAC as compared to BO. This would agree with the hypothesis.

6. A discussion should be made of why of the analyses, only MYBL2 (Figure 7D) related to survival whereas JUP and CCNE did not (Figure S9C).

*Reviewer #2 (Recommendations for the authors):*

There is a significant amount of new CUT&TAG data, and it seems that this could have been more clearly presented. For example, although Figure 2C shows a correlation between different marks, this figure does not show how these marks correlate with eRNA regions compared to randomly accessible regions. Figure S2C shows this, but comparison is not quantitative, and not all marks are included.

Comparisons of eRNA regions with promoters in S2D-G are not useful, and I would suggest they be omitted. It is a biased analysis, as promoters are intentionally excluded from the eRNA identification.

In the text discussing Figure 2D, the authors point out that 43% of eRNA are mapped to enhancer regions, but do not give the comparative context that 38% of random open regions map to enhancers, which makes the finding somewhat underwhelming. In contrast, the authors do not mention the largest enrichment in eRNA regions – Active TSS (18% versus 12%). This seems due to the presence of the active TSS that makes the eRNA, and is quite interesting.

The discussion states "Importantly, our enhancer repertoire identified new pathways that are activated in OAC which were not apparent from either genome sequencing or mRNA profiling alone". This statement is not well supported by the data. It is not clear from the manuscript what these new pathways are.

*Reviewer #3 (Recommendations for the authors):*

1) The authors compare regions expressing eRNAs in AOC patient samples with epigenetic and transcription factor binding profiles in AOC-derived OE19 cells. They insist on the regions that converge from both analyses. While this is important to validate the approach, they seem to have excluded eRNA regions specific to patient samples. OE19 cells are obviously adapted to growth in culture, but it should be informative to focus also on eRNAs and associated genes that are found only in patient samples as this may provide further insights into genes that are important for in situ tumour growth as opposed to those involved in growth of OE19 cells in culture. In particular, the role of these regions would be strengthened if they were also associated with KLF5 of other TFs shown to be important in OAC.

2) It is not clear to this referee why the overlap between differentially expressed eRNAs and differentially expressed genes is so low as illustrated in Figure 4B. Is it correct to interpret these results to mean that there are multiple eRNAs that show differential expression, but no change in the associated genes, whereas the vast majority of differentially expressed genes showed no associated eRNAs. Also, most of the ontology terms identified by eRNA-associated genes and differentially expressed genes are in fact different, while the authors imply they are similar. Can the authors comment on this?

3) Some of the data in Figure 5 is not very convincing. For example, the enhancer activity measured by the pGL3 vectors is very weak, whereas it is much more convincing with the STARR vectors. The pGL3 data could be removed. Also, can the authors express the data in 5D as fold change over the control vector rather than 'reporter expression'. Similarly, the changes in JUP, CCNE1 and MYBL2 in panel E are rather weak compared to the changes in the corresponding eRNAs. Given the extent of these changes, it is difficult to understand the strong effects on viability in Figure 7B. Can the authors comment?

4) From the text, it is not clear how the authors defined the 6-gene signature that was used to associate with OS in Figure 7E this should be better described (there is a description in the Methods section, but it could be made clearer). Also, can the authors demonstrate that a signature based on differentially expressed eRNA-associated genes has better clinical value than signatures based on differentially expressed genes. As mentioned above the data in Figure 4 shows only limited overlap. For example, do the 59 overlapping AOC genes in Figure 4B allow better association with clinical outcome than signatures based on the most differentially expressed genes or the 340 most differentially expressed eRNA-associated genes? Is the 6-gene signature in Figure 7E present in this list?

# Reply: Author response

**DOI:** 10.7554/eLife.80840.sa2

Essential revisions:

1) The authors discuss AP1, KLF5, and HNF1 in Figure 3. However, they have not demonstrated that eRNAs specific to OACs also relate to their ability to transcribe target genes. They should consider if these TFs regulate JUP based as a function of JUPe expression. As one example, upon CRISPRi of JUPe, they should determine that these factors have diminished regulatory activity of JUP transcripts through ChIP-qPCR coupled with the existing RT-PCR experiments.

We agree this is an important point and have added extra experimental data to support a link between enriched transcription factors and target enhancer/gene activity. We focussed on KLF5 to provide functional links between KLF5 occupancy at enhancers, enhancer activation and target gene transcription. Our previously published ChIP-seq data demonstrate that KLF5 strongly binds to the *JUP* and *CCNE1* enhancers but very low levels at the MYBL2 enhancer (new Figure 3G). KLF5 depletion caused reduced levels of all target genes (new Figure 3H). However only *JUP* and *CCNE1* enhancer activities was diminished following KLF5 depletion (new Figure 3I), consistent with the higher occupancy of KLF5 at these enhancers compared to *MYBL2*. *MYBL2* is likely regulated by KLF5 though other cis regulatory elements or by an indirect mechanism. Our data are therefore consistent with KLF5 regulating *JUP* and *CCNE1* expression through the enhancers we have identified.

2) Enhancers may regulate transcript of more than one gene. The authors should consolidate the relationship between eRNA expression of JUP, CCNE1, and MYBL2, based on structural interactions. A potential experiment is Hi-C sequencing.

In the paper we already show that these enhancers do not influence the activity of other nearby genes. However, as requested by the reviewer, we have now performed Hi-C in OE19 cells to attempt to identify any linkages between these enhancers and other more distally located genes. We were unable to discover any new linkages for *CCNE1*e and *MYBL2*e enhancers. However, we were able to identify new linkages for the JUPe enhancer (new Figure 6E) where it links close to the *ERBB2* locus. Downregulation of enhancer activity does not affect *ERBB2* expression and the neighbouring *MIEN1* gene but does affect neighbouring *GRB7* expression (new Figure 6F) and both the *JUP*e and *GRB7* are sensitive to KLF5 depletion (new Figure 6—figure supplement 2C).

3) From the text, it is not clear how the authors defined the 6-gene signature that was used to associate with OS in Figure 7E this should be better described (there is a description in the Methods section, but it could be made clearer). Also, can the authors demonstrate that a signature based on differentially expressed eRNA-associated genes has better clinical value than signatures based on differentially expressed genes? As mentioned above the data in Figure 4 shows only limited overlap. For example, do the 59 overlapping AOC genes in Figure 4B allow better association with the clinical outcome than signatures based on the most differentially expressed genes or the 340 most differentially expressed eRNA-associated genes? Is the 6-gene signature in Figure 7E present in this list?

We clarified the text in the Materials and methods. We also performed the additional analyses requested by the reviewer (new Figure 7—figure supplement 1F). In all cases, we could derive prognostic signatures, although different genes/loci contributed to each case. This is illustrated by the fact that only 3 of the DEE associated signature genes in Figure 7E also represent DEGs. We therefore conclude that DEEs able to predict prognostic signatures on their own to an equivalent level as using DEGs.

4) Some of the data in Figure 5 is not very convincing. For example, the enhancer activity measured by the pGL3 vectors is very weak, whereas it is much more convincing with the STARR vectors. The pGL3 data could be removed. Also, can the authors express the data in 5D as fold change over the control vector rather than 'reporter expression'. Similarly, the changes in JUP, CCNE1, and MYBL2 in panel E are rather weak compared to the changes in the corresponding eRNAs. Given the extent of these changes, it is difficult to understand the strong effects on viability in Figure 7B. Can the authors comment?

Although the luciferase reporter assay results are less striking than the data from the STARR vector assays, they do provide important independent validation. We have therefore retained these but moved to the supplementary figures. As requested we now express the data from the STARR reporters in Figure 6C as expression relative to the negative control.

The reduced effect on target gene expression compared to enhancer transcription is not unexpected as the target genes are likely controlled by many elements including promoter proximal elements. We have commented on this in the text. Perhaps more surprisingly are the strong phenotypic effects we see and could be explained either by there being a particular dose-sensitive response to these genes or alternatively the surviving cells may have found ways to engage different regulatory elements to circumvent the detrimental effects of reduced expression. Furthermore, although our newly added HiC data do not uncover major linkages to other more distal genes (with the exception of *JUP*- see discussion of point 2 above) it remains possible that the enhancers control the activity of other genes needed for cell viability. We have added further discussion of these possibilities and potential limitations as requested.

5) There is a significant amount of new CUT&TAG data, and it seems that this could have been more clearly presented. For example, although Figure 2C shows a correlation between different marks, this figure does not show how these marks correlate with eRNA regions compared to randomly accessible regions. Figure S2C shows this, but the comparison is not quantitative, and not all marks are included.

We have now added all of the heatmaps to Figure 2—figure supplement 1C &D as requested and also included Average tag density plots to demonstrate the increased signal associated with eRNA regions for a range of enhancer associated histone marks and a depletion of promoter associated marks.

6) The authors should put the context of the percentages of eRNAs found at enhancers and other regions to make it clear which findings are significant.

We re-evaluated this data and found an error in HMM assignments. Also, we used a different background dataset for comparison than the original accessible genome and used the whole genome as the aim was to illustrate that we were identifying enhancer regions rather than random pieces of chromatin. This new data in Figure 2—figure supplement 1J clearly indicates an enrichment of enhancer-like regions (33% vs 4%) rather than quiescent/repressed regions (18% vs 77%) in our eRNA-defined genomic regions.

Reviewer #1 (Recommendations for the authors):

1. The authors discuss AP1, KLF5, and HNF1 in Figure 3. However, they have not demonstrated that eRNAs specific to OACs also relate to their ability to transcribe target genes. They should consider if these TFs regulate JUP based as a function of JUPe expression. As one example, upon CRISPRi of JUPe, they should determine that these factors have diminished regulatory activity of JUP transcripts through ChIP-qPCR coupled with the existing RT-PCR experiments.

We agree this is an important point and have added extra experimental data to support a link between enriched transcription factors and target enhancer/gene activity. We focussed on KLF5 to provide functional links between KLF5 occupancy at enhancers, enhancer activation and target gene transcription. Our previously published ChIP-seq data demonstrate that KLF5 strongly binds to the *JUP* and *CCNE1* enhancers but very low levels at the MYBL2 enhancer (new Figure 3G). KLF5 depletion caused reduced levels of all target genes (new Figure 3H). However only *JUP* and *CCNE1* enhancer activities was diminished following KLF5 depletion (new Figure 3I), consistent with the higher occupancy of KLF5 at these enhancers compared to *MYBL2*. *MYBL2* is likely regulated by KLF5 though other cis regulatory elements or by an indirect mechanism. Our data are therefore consistent with KLF5 regulating *JUP* and *CCNE1* expression through the enhancers we have identified.

2. Enhancers may regulate the transcript of more than one gene. The authors should consolidate the relationship between eRNA expression of JUP, CCNE1, and MYBL2, based on structural interactions. A potential experiment is Hi-C sequencing.

In the paper we already show that these enhancers do not influence the activity of other nearby genes. However, as requested by the reviewer, we have now performed Hi-C in OE19 cells to attempt to identify any linkages between these enhancers and other more distally located genes. We were unable to discover any new linkages for *CCNE1*e and *MYBL2*e enhancers. However, we were able to identify new linkages for the JUPe enhancer (new Figure 6E) where it links close to the *ERBB2* locus. Downregulation of enhancer activity does not affect *ERBB2* expression and the neighbouring *MIEN1* gene but does affect GRB7 expression (new Figure 6F) and both the *JUP*e and *GRB7* are sensitive to KLF5 depletion (new Figure 6—figure supplement 2C).

3. Based on the same concerns, in Figure 6E – the transcript expression suppressing effects of targeting the eRNA is small when examining JUP, CCNE1, and MYBL2, despite robust suppression of the eRNAs. However, this also corresponds to a significant viability effect in Figure 7B. The concern is that the eRNA represents a measure of regulatory activity on more than one target gene and that other targets also contribute to the viability regulating phenotypes. The authors should demonstrate the specificity of the experiments. As one example, related to JUPe, authors should examine other genes on 17q, such as ERBB2. A key demonstration would be that these 17q genes are not associated with JUPe in OAC samples, that JUPe suppression does not impact their expression in cells, and that suppression of these other 17q genes does not have viability effects in the same set of cell lines. Based on Hi-C experiments, they should also confirm there is no interaction with ERBB2 or other genes.

This is covered in our responses to points 1 and 2 above.

4. Outside of JUPe, the authors have not examined if JUP has general roles in OACs without ERBB2 amplifications. In Figure 7A, the cell line results from Depmap include 6 ERBB2 amplified models. In Figure S7C there are no OACs with JUP amplification outside of samples with ERBB2 amplification. This coincides with the lack of OS in Figure S9C.

The referee raises a good point and we suspect that the JUP dependency likely relates to the *ERBB2* amplified tumours. However, the numbers of tumours available with ERBB2 plus/minus JUP preclude making any definitive conclusions. However, we agree with the reviewer about the implications for why JUP is not generally related to patient survival and added a comment to the results text about this when discussing Figure 7—figure supplement 1D.

5. A concern is that JUPe measurements are a surrogate for cell activity that is not specific to JUP expression. In Figure 5C, JUPe is not expressed in many samples that have clear JUP expression, which suggests JUPe is not essential for JUP expression. This figure panel is also hard to resolve since BO and OAC samples are in the same plot. The authors should consider splitting Figure 5C into two panels to demonstrate that JUP expression is tightly regulated by JUPe in OAC as compared to BO. This would agree with the hypothesis.

The lack of *JUPe* expression where there is high *JUP* expression is likely a technical issue where eRNAs are lost in the particular samples (note here we are looking at total RNA levels and unstable eRNAs may not always be detectable in RNAseq datasets from patient samples). As requested, we have added an additional panel to Supplementary Figure S7 (new Figure 5—figure supplement 1G) to depict the relationship between *JUPe* expression and *JUP* coding transcript expression when segregating the BO and OAC samples and analysing them separately. This new data further supports our hypothesis as the correlation between *JUPe* and *JUP* expression is even higher when considering just the OAC samples.

6. A discussion should be made of why of the analyses, only MYBL2 (Figure 7D) related to survival whereas JUP and CCNE did not (Figure S9C).

We have dealt with the discussion surrounding *JUP* in response to point 4 above. It is not clear why *CCNE1* does not relate to overall survival but might reflect an early essential event for tumour formation, which would not be unexpected for a core cell cycle gene. We do not think it is warranted to add further discussion of *CCNE1* and overall survival.

Reviewer #2 (Recommendations for the authors):

There is a significant amount of new CUT&TAG data, and it seems that this could have been more clearly presented. For example, although Figure 2C shows a correlation between different marks, this figure does not show how these marks correlate with eRNA regions compared to randomly accessible regions. Figure S2C shows this, but comparison is not quantitative, and not all marks are included.

We have now added all of the heatmaps to Figure 2—figure supplement 1C &D as requested and also included Average tag density plots to demonstrate the increased signal associated with eRNA regions for a range of enhancer associated histone marks and a depletion of promoter associated marks.

Comparisons of eRNA regions with promoters in S2D-G are not useful, and I would suggest they be omitted. It is a biased analysis, as promoters are intentionally excluded from the eRNA identification.

We agree that this is a biased analysis but is deliberately so as we are trying to demonstrate that the eRNA regions resemble enhancer regions and not promoter regions as defined by chromatin marks (which are not gathered in a biased manner). As such, these represent control comparisons to validate the enhancer designations rather than a random association with all chromatin marks. This in part also validates our CUT&TAG data and as such we believe is useful to retain in the paper.

In the text discussing Figure 2D, the authors point out that 43% of eRNA are mapped to enhancer regions, but do not give the comparative context that 38% of random open regions map to enhancers, which makes the finding somewhat underwhelming. In contrast, the authors do not mention the largest enrichment in eRNA regions – Active TSS (18% versus 12%). This seems due to the presence of the active TSS that makes the eRNA, and is quite interesting.

We re-evaluated this data and found an error in HMM assignments. Also, we used a different background dataset for comparison than the original accessible genome and used the whole genome as the aim was to illustrate that we were identifying enhancer regions rather than random pieces of chromatin. This new data in Figure 2—figure supplement 1J clearly indicates an enrichment of enhancer-like regions (33% vs 4%) rather than quiescent/repressed regions (18% vs 77%) in our eRNA-defined genomic regions. We agree that in the original paper the TSS enrichment is potentially interesting but might reflect regions with the potential to act as enhancers and promoters. However as observed in Figure 2—figure supplement 1D very few regions seem to exhibit strong promoter activity as defined by H3K4me3 levels, and the HMM designations would not provide such a granularity in promoter strength. This is an open question that needs more investigation and we have not discussed this further.

The discussion states "Importantly, our enhancer repertoire identified new pathways that are activated in OAC which were not apparent from either genome sequencing or mRNA profiling alone". This statement is not well supported by the data. It is not clear from the manuscript what these new pathways are.

We have changed the wording to “"Importantly, our enhancer repertoire identified new molecular events that have been activated in OAC…”. Further discussion of these events and the linked pathways is in paragraph 3 of the discussion where connections to the underlying data are provided.

Reviewer #3 (Recommendations for the authors):

1) The authors compare regions expressing eRNAs in AOC patient samples with epigenetic and transcription factor binding profiles in AOC-derived OE19 cells. They insist on the regions that converge from both analyses. While this is important to validate the approach, they seem to have excluded eRNA regions specific to patient samples. OE19 cells are obviously adapted to growth in culture, but it should be informative to focus also on eRNAs and associated genes that are found only in patient samples as this may provide further insights into genes that are important for in situ tumour growth as opposed to those involved in growth of OE19 cells in culture. In particular, the role of these regions would be strengthened if they were also associated with KLF5 of other TFs shown to be important in OAC.

We used epigenetic marks from OE19 cells to validate our findings. However, the eRNAs we take forwards in Figure 3 onwards represent all eRNAs, not just those mapping to OE19 accessible regions defined by ATAC-seq. Nevertheless, the referee makes a good point and we have analysed the eRNA-defined enhancer regions that are not in OE19 and are just found in patients (ie a subset of what we analysed previously). As observed for the total dataset, the top three transcription factor binding motifs are among the top motifs in the patient specific eRNA-defined enhancer regions (TEAD3, AP-1 and CTCF; compare Figure 3A with new Figure 3—figure supplement 1A). Very similar GO terms are also returned as significant in both cases (compare Figure 4A and Figure 3—figure supplement 1I). “Cell adhesion” is revealed as a category that appears only for the patient-specific eRNA-associated genes, which may reflect the differences between 2D culture and the in vivo environment. We added discussion of this new data to the manuscript.

2) It is not clear to this referee why the overlap between differentially expressed eRNAs and differentially expressed genes is so low as illustrated in Figure 4B. Is it correct to interpret these results to mean that there are multiple eRNAs that show differential expression, but no change in the associated genes, whereas the vast majority of differentially expressed genes showed no associated eRNAs. Also, most of the ontology terms identified by eRNA-associated genes and differentially expressed genes are in fact different, while the authors imply they are similar. Can the authors comment on this?

There are many possible reasons for the overall lack of overlap in differential expression of eRNAs and their associated gene expression. The first is that the nearest gene model may not always be the most appropriate linkage. Secondly, not all genes are necessarily driven by enhancers and/or a change in enhancer activity rather than promoter activity. Thirdly, the threshold cut-offs for calling differential expression may preclude associations. Fourth, we are also likely missing many eRNAs due to the datasets we used which are not designed for eRNA identification. Finally, it is possible that as one enhancer becomes activated, another becomes inactivated, so there is no net change in gene expression and yet enhancer activity changes significantly. The latter possibility was already discussed in the paper and we have now added further discussion for the lack of congruency. Nevertheless, the associations we do find between differentially expressed eRNAs and associated targets are significant.

The referee makes a good point about the GO terms and we have rephrased the text describing these. However, the majority are very similar in OAC ie KRAS signalling and MAPK signalling are the same pathway (and relate to MAPK signalling), ECM organisation and EMT are metastatic like properties (and relate to cell migration) and inflammatory signalling relates to cytokine production. We have however now pointed out that embryonic development and histone methylation are new terms revealed by eRNA signatures which further emphasises the new information this analysis has provided.

3) Some of the data in Figure 5 is not very convincing. For example, the enhancer activity measured by the pGL3 vectors is very weak, whereas it is much more convincing with the STARR vectors. The pGL3 data could be removed. Also, can the authors express the data in 5D as fold change over the control vector rather than 'reporter expression'. Similarly, the changes in JUP, CCNE1 and MYBL2 in panel E are rather weak compared to the changes in the corresponding eRNAs. Given the extent of these changes, it is difficult to understand the strong effects on viability in Figure 7B. Can the authors comment?

Although the luciferase reporter assay results are less striking than the data from the STARR vector assays, they do provide important independent validation. We have therefore retained these but moved to the supplementary figures. As requested we now express the data from the STARR reporters in Figure 6C as expression relative to the negative control.

The reduced effect on target gene expression compared to enhancer transcription is not unexpected as the target genes are likely controlled by many elements including promoter proximal elements. We have commented on this in the text. Perhaps more surprisingly are the strong phenotypic effects we see and could be explained either by there being a particular dose-sensitive response to these genes or alternatively the surviving cells may have found ways to engage different regulatory elements to circumvent the detrimental effects of reduced expression. Furthermore, although our newly added HiC data do not uncover major linkages to other more distal genes (with the exception of *JUP*- see discussion of point 2 above) it remains possible that the enhancers control the activity of other genes needed for cell viability. We have added further discussion of these possibilities as requested.

4) From the text, it is not clear how the authors defined the 6-gene signature that was used to associate with OS in Figure 7E this should be better described (there is a description in the Methods section, but it could be made clearer). Also, can the authors demonstrate that a signature based on differentially expressed eRNA-associated genes has better clinical value than signatures based on differentially expressed genes. As mentioned above the data in Figure 4 shows only limited overlap. For example, do the 59 overlapping AOC genes in Figure 4B allow better association with clinical outcome than signatures based on the most differentially expressed genes or the 340 most differentially expressed eRNA-associated genes? Is the 6-gene signature in Figure 7E present in this list?

We clarified the text in the Materials and methods. We also performed the additional analyses requested by the reviewer (new Figure 7—figure supplement 1F). In all cases, we could derive prognostic signatures, although different genes/loci contributed to each case. This is illustrated by the fact that only 3 of the DEE associated signature genes in Figure 7E also represent DEGs. We therefore conclude that DEEs able to predict prognostic signatures on their own to an equivalent level as using DEGs.
